# Genetic variability and population structure within the *Anopheles tessellatus* complex (Theobald, 1901) in Indonesia using ITS2 nuclear and COI, COII mitochondrial sequences

**DOI:** 10.1371/journal.pone.0347730

**Published:** 2026-08-14

**Authors:** Anis Nurwidayati, Hari Purwanto, Raden Roro Upiek Ngesti Wibawaning Astuti, Yudhi Ratna Nugraheni, Lulus Susanti, Yuyun Srikandi, Budi Setiadi Daryono, Triwibowo Ambar Garjito, Sylvie Manguin

**Affiliations:** 1 School of Doctoral Biology, Faculty of Biology, Universitas Gadjah Mada, Jl. Teknika Selatan, Sekip Utara, Yogyakarta, Indonesia; 2 Laboratory of Disease Vector and Reservoir, Research Center for Public Health and Nutrition, National Research and Innovation Agency (BRIN), Jl. Hasanudin 123A, Salatiga, Central Java, Indonesia; 3 Laboratory of Entomology, Faculty of Biology, Universitas Gadjah Mada, Jl. Teknika Selatan, Sekip Utara, Yogyakarta, Indonesia; 4 Parasitology Division, Laboratory of Animal Systematics, Faculty of Biology, Universitas Gadjah Mada, Jl. Teknika Selatan, Sekip Utara, Yogyakarta, Indonesia; 5 Department of Parasitology, Faculty of Veterinary Medicine, Universitas Gadjah Mada, Jl. Fauna No.2, Karang Gayam, Depok, Yogyakarta, Indonesia; 6 Environmental Health Laboratory Center Salatiga, Jl. Hasanudin No 123, Salatiga, Central Java, Indonesia; 7 Public Health Laboratory Center, Donggala, Jl. Masitudju No 58, Donggala, Central Sulawesi, Indonesia; 8 Laboratory of Genetics and Breeding, Faculty of Biology, Universitas Gadjah Mada, Jl. Teknika Selatan, Sekip Utara, Yogyakarta, Indonesia; 9 HSM, Univ. Montpellier, CNRS, IRD, Montpellier, France; Instituto Leonidas e Maria Deane / Fundacao Oswaldo Cruz, BRAZIL

## Abstract

Some *Anopheles* species that act as malaria vectors are members of species complexes, a concept whereby sibling species cannot be differentiated solely on the basis of morphological characters. Therefore, species complexes represent a major problem in malaria vector control, because within an *Anopheles* complex, vectors cannot be differentiated from non-vector species, unless molecular techniques are used to identify them. The *Anopheles tessellatus* species complex is an important potential vector in South, East, and Southeast Asia, including certain regions of Indonesia. However, no in-depth studies have been conducted on this species complex in that country. Therefore, this study investigated the taxonomic status of *An. tessellatus* from diverse populations across five Indonesian islands (Sumatra, Java, West Nusa Tenggara (WNT), East Nusa Tenggara (ENT), and Sulawesi) and identified interpopulation genetic variation based on molecular data of the ITS2, COI, and COII genes. Phylogenetic relationships were constructed using the Maximum Likelihood method. Haplotype and network analysis were also conducted. The results indicate that *An. tessellatus* constitutes a monophyletic group comprising three well‑defined lineages that exhibit clear intraspecific genetic differentiation. Cluster 1 corresponds to the population of Sumatra, Cluster 2 represents the population from Sulawesi, and Cluster 3 encompasses populations from Java, West Nusa Tenggara, and East Nusa Tenggara. These findings demonstrate high haplotype diversity and low nucleotide diversity within the species. Populations from West Sumatra, Manado, Tojo Una-Una, and North Morowali (Sulawesi) revealed strong population structure within a single species with genetic distances of 0.61 – 0.94% for COI, between 0.81 – 0.95% for ITS2, and between 0.62 – 0.71% for COII. These findings underscore the need for further integrative studies to obtain a more comprehensive understanding of the *An. tessellatus* complex in Indonesia and its role in malaria transmission.

## 1. Introduction

*Anopheles tessellatus* (Theobald,1901) is recognized as a potentially significant vector of malaria and lymphatic filariasis across South, East, and Southeast Asia [1]. This taxon has been identified as the primary vector of pathogens responsible for these diseases in regions such as the Maldives, Sri Lanka, and certain parts of Indonesia. Taxonomically, the Tessellatus Group belongs to the Neomyzomyia Series of the *Anopheles* subgenus *Cellia.* It includes two species: *An. orientalis* and *An. tessellatus*, as well as the Tessellatus complex with six unnamed species A, B, C, D, E, and F [2,3]. Originally described from specimens collected in Taiping, Perak, in Northwest Peninsular Malaysia [4], this species exhibits considerable complexity throughout Southeast Asia [2]. The taxonomic history of *An. tessellatus* is notably intricate, characterized by marked morphological diversity among populations, leading to the recognition of several synonyms and subspecies, particularly in Indonesia [5].

Morphological analyses have identified at least 11 morphological variants of *An. tessellatus* within the Indonesian archipelago. Among these, three subspecies have been formally recognized: *An. tessellatus tessellatus* (Theobald,1901); *An. tessellatus kalawara* (Stoker & Koesoemawinangoen, 1949), both described from Indonesia, and *An. tessellatus orientalis* (Swellengrebel and de Graaf, 1920), which is distributed across Sulawesi, Java, and the Maluku Islands [5]. The observed morphological diversity suggests the possible presence of cryptic lineages within the Tessellatus Complex, as recent molecular studies using COI gene analyses have identified up to six putative species within what was previously considered a single taxon [2]. Such cryptic diversity has significant implications for disease transmission dynamics and vector management, as divergent lineages may differ in their vectorial capacity and ecological characteristics [2].

Despite its epidemiological relevance and taxonomic complexity, studies on the genetic variation and population structure of *An. tessellatus* in Indonesia remains limited. Traditional morphological identification is often insufficient due to the isomorphic nature of sibling species, making molecular markers essential for accurate species delimitation and population genetic studies [6]. Among these, the Internal Transcribed Spacer 2 (ITS2) region of ribosomal DNA and the mitochondrial cytochrome c oxidase subunit I (COI) and subunit II (COII) genes have proven to be reliable tools for distinguishing closely related *Anopheles* species and assessing their genetic diversity [7]. Both ITS2 and COI markers have been widely used in population genetics, phylogenetic, and phylogeographic studies of *Anopheles* mosquitoes across Asia, revealing significant intra- and interspecific variation and uncovering cryptic species complexes [8]. Various molecular studies of *Anopheles* mosquitoes have also used the COI gene, including to identify members of the species complex. One example is the identification of the *An. subpictus* complex in Sri Lanka [9]. Studies on the genetic variation and population structure of *An. subpictus, An. peditaeniatus,* and *An. vagus* mosquitoes from five regions in Sri Lanka were also studied using the COI marker gene [6]. The COI gene, in particular, has been a cornerstone in studies addressing population structure and systematics of *Anopheles* mosquitoes [10,11].

Previous studies have demonstrated the effectiveness of COI and ITS2 markers in identifying both Culicinae and *Anopheles* mosquitoes in diverse geographic settings, including Sri Lanka [6]. The ITS2 marker has been employed to elucidate species diversity and relationships among *Anopheles* populations in Malaysia, along the Laos-Cambodia border [12], where novel species within the *An. hyrcanus* group [13] and *An. lesteri* in Korea were discovered [14]. Similarly, investigations of *An. anthropophagus* populations in China have relied on ITS2 for species confirmation [12,14] and a novel ITS2-based PCR was recently developed to better identify members of the *An. dirus* complex [15]. In addition to ITS2 and COI, the mitochondrial cytochrome c oxidase subunit II (COII) gene has emerged as a promising molecular marker for species identification and population genetic studies. For instance, studies on the *An. culicifacies* complex have successfully applied ITS2 and COII markers to resolve species boundaries [16]. Moreover, combined analyses of COI and COII genes have provided insights into the genetic population structure of *An. superpictus* in Iran [17].

Given these considerations, the present study aims to investigate the interpopulation genetic variation and population structure of *An. tessellatus* across Indonesia using molecular data from three genetic markers: the nuclear ITS2 ribosomal gene and the mitochondrial COI and COII genes. Specimens were collected from six geographically distinct populations representing Sumatra, Java, Kalimantan, the Lesser Sunda Islands, Sulawesi, and Papua. This thorough molecular approach is expected to provide a more comprehensive understanding of the genetic diversity, evolutionary relationships, and potential vectorial capacity of *An. tessellatus* in Indonesia, thereby contributing valuable information for malaria and lymphatic filariasis control programs in the studied areas of Indonesia.

## 2. Methods

### 2.1. Sample collections and identification

**Fig 1:**
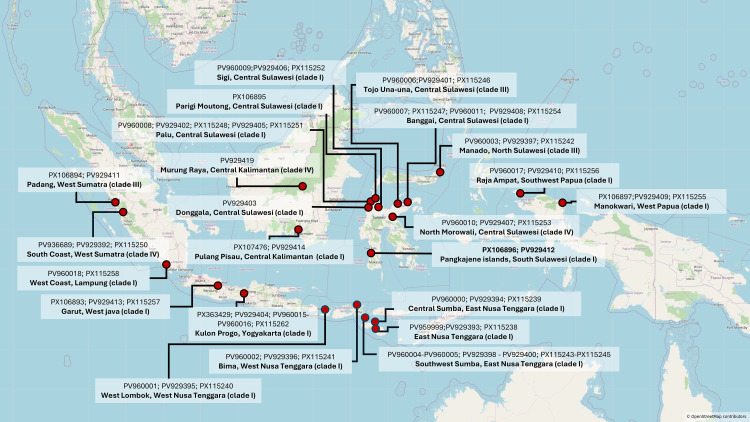
Distribution map of *Anopheles tessellatus* sampling locations in Indonesia, marked with red dots.

Adult mosquitoes were collected from diverse field environments across 13 Indonesian provinces between 2015 and 2024, employing standardized human landing catches, cattle-bait collections, US-CDC light traps, and animal-baited trap techniques. Morphological identification of *Anopheles tessellatus* was performed using the illustrated adult mosquito identification key for Indonesia by O’Connor and Soepanto (1999). Sampling sites encompassed the South Coast (Pesisir Selatan) in West Sumatra; Central Sumba and Southwest Sumba in East Nusa Tenggara; West Lombok and Bima in West Nusa Tenggara; Manado in North Sulawesi; Tojo Una-Una, Banggai, North Morowali, Sigi, Palu, and Donggala in Central Sulawesi; West Coast (Pesisir Barat) in Lampung; Murung Raya in Central Kalimantan; and Kulon Progo in the Special Region of Yogyakarta. (Fig 1; Table 1). Initial identification of *An. tessellatus* specimens were performed based on morphological characteristics. Collected mosquitoes were sorted and labelled by location and collection date, then preserved in 1.5 ml Eppendorf tubes containing silica gel to maintain dry conditions until further analysis [18]. Samples were obtained from field mosquito collections, and access to stored biological materials (*Anopheles* *barbirostris* mosquitoes) was obtained from the Center for Environmental Health Laboratory of the Ministry of Health, Indonesia. Permissions to access these samples for research purposes were secured via a Material Transfer Agreement authorized by the Director of the Center for Environmental Health Laboratory (no. HK.03.01/IX.1/3787/2024) and the Dean of the Faculty of Biology, Universitas Gadjah Mada (no. 6941/UN1/FBI/KSA/HK.08.00/2024) on December 14, 2024.

**Table 1:** Sampling localities and specimens of the *Anopheles tessellatus* complex (Number sequenced).

Sample code	Province	Location	Number of sample and Accession number					
			COI		ITS2		COII	
01	West Sumatra	South Coast (Pesisir Selatan)	1	PV936689	1	PV929392	1	PX115250
02	East Nusa Tenggara	Pooling (unknown)	1	PV959999	1	PV929393	1	PX115238
05	East Nusa Tenggara	Central Sumba	1	PV960000	1	PV929394	1	PX115239
09	West Nusa Tenggara	West Lombok	1	PV960001	1	PV929395	1	PX115240
10	West Nusa Tenggara	Bima	1	PV960002	1	PV929396	1	PX115241
12	North Sulawesi	Manado	1	PV960003	1	PV929397	1	PX115242
14	East Nusa Tenggara	Southwest Sumba - Pandawawi	1	PV960004	1	PV929398	1	PX115243
15	East Nusa Tenggara	Southwest Sumba - Matakapore	1	PV960005	1	PV929399	1	PX115244
16	East Nusa Tenggara	Southwest Sumba - Galukalogo	0	-	1	PV929400	1	PX115245
17	Central Sulawesi	Tojo Una-Una	1	PV960006	1	PV929401	1	PX115246
18	Central Sulawesi	Banggai 1 - Nuhon	1	PV960007	0	-	1	PX115247
20	Central Sulawesi	Palu 1 - Mantikulore	1	PV960008	1	PV929402	1	PX115248
25	Central Sulawesi	Donggala	0	-	1	PV929403	0	-
27	Yogyakarta	Kulon Progo 1	1	PX363429	1	PV929404	0	-
35	Central Sulawesi	Palu 2 - Mantikulore	0	-	1	PV929405	1	PX115251
36	Central Sulawesi	Sigi-Sunju	1	PV960009	1	PV929406	1	PX115252
37	Central Sulawesi	North Morowali	1	PV960010	1	PV929407	1	PX115253
38	Central Sulawesi	Banggai 2 - Nuhon	1	PV960011	1	PV929408	1	PX115254
39	West Papua	Manokwari	1	PX106897	1	PV929409	1	PX115255
42	Southwest Papua	Raja Ampat	1	PV960017	1	PV929410	1	PX115256
43	West Sumatra	Padang	1	PX106894	1	PV929411	0	-
46	South Sulawesi	Pangkajene Islands	1	PX106896	1	PV929412	0	-
47	West Java	Garut	1	PX106893	1	PV929413	1	PX115257
50	Central Kalimantan	Pulang Pisau	1	PX107476	1	PV929414	0	-
53	Lampung	West Coast (Pesisir Barat) 1	1	PV960018	0	-	1	PX115258
87	Yogyakarta	Kulon Progo 5	1	PV960015	0	-	1	PX115262
88	Yogyakarta	Kulon Progo 6	1	PV960016	0	-	0	-
85	Central Sulawesi	Parigi Moutong	1	PX106895	0	-	0	-
102	Central Kalimantan	Murung Raya	0	-	1	PV929419	0	-
	**Total**		**25**		**24**	PV929392	**21**	

### 2.2. DNA extraction and PCR amplification

Genomic DNA was isolated from individual mosquito samples using the Zymo Quick-DNA Miniprep Plus DNA extraction kit (ZYMO, Irvine, CA, USA) according to the manufacturer’s protocols. Three genetic loci were targeted for PCR amplification: the internal transcribed spacer 2 (ITS2) region, cytochrome c oxidase subunit I (COI), and cytochrome c oxidase subunit II (COII). ITS2 was amplified with primers ITS2a (5’-TGTGAACTGCAGGACACAT-3’) and ITS2b (5-TATGCTTAAATTCAGGGGGT-3’), COI with primers LCO1490 (5’-GGTCAACAAATCATAAAG ATATTGG-3’) and HCO2198 (5’-AAACTTCAGGGTGACCAAAAAATCA-3’), and COII with primers Anplcox2F (5’-GGATCCAGATTAGTGCAATGAATTTAAGC-3’) and Anplcox2R (5’-CTGCAGGATTTAAGAGATCATACTTGC-3’). PCR reactions were performed using GoTaq Green Master Mix (Promega, Madison, WI, USA). Thermocycling conditions for ITS2 consisted of an initial denaturation at 94 °C for 10 min, followed by 40 cycles of 94 °C for 1 min, 52 °C for 45 s, and 72 °C for 1 min, with a final extension at 72 °C for 10 min. For COI, the protocol included an initial denaturation at 94 °C for 1 min; five cycles of 94 °C for 30 s, 45 °C for 40 s, and 72 °C for 1 min; followed by 35 cycles of 94 °C for 30 s, 55 °C for 40 s, and 72 °C for 1 min; and a final extension at 72 °C for 10 min. COII amplification followed the same cycling parameters as COI, except the annealing temperature during the 35 cycles was set at 58 °C. PCR products were separated by electrophoresis on 1.5% agarose gels, visualized using SYBR Safe DNA gel stain (Invitrogen, Carlsbad, CA, USA), and fragment sizes were estimated with a 100 bp DNA ladder.

### 2.3. DNA sequencing and data analysis

PCR amplification products were then purified using the ExoSAP-IT™ reagent (Applied Biosystems, Thermo Fisher Scientific, Vilnius, Lithuania) prior to sequencing. Sequencing reactions were performed using the same primers described above, with the BigDye^TM^ Terminator v3.1 Cycle Sequencing Kit (Life Technologies Corporation, Austin, TX, USA). Resulting sequences were processed and edited using Sequencing Analysis software version 5.2 (Applied Biosystems). After PCR amplification and sequencing, 24 ITS, 25 COI, and 21 COII sequences were obtained and included in downstream analyses.

Multiple sequence alignments were generated with ClustalW 1.6 implemented in MEGA X version 10.2.2. The obtained sequences were compared against reference sequences of *Anopheles tessellatus* available in the NCBI GenBank database. Multiple sequence alignment was performed using MUSCLE v.5.3 [19]. The aligned sequences were further trimmed with trimAl v.1.5 [20] using the “automated” setting, which was optimized for Maximum Likelihood (ML) phylogenetic tree reconstruction. ML trees were inferred using IQ-TREE v.2.3.6 [21] with optimal models selected from the full range of models supported by ModelFinder [22]. Clade support was assessed with 1,000 bootstrap replicates using UFBoot2 [23]. Hierarchical cluster analysis was performed to identify clusters in the reconstructed phylogenetic trees using R v.4.2.2 [24]. Pairwise p-distance distances were calculated using the APE R package [25]. The minimum interspecific and maximum intraspecific distances were calculated using the SPIDER R package [26]. The standard genetic indices, haplotype diversity (h), haplotype number (H), and nucleotide diversity (π) were evaluated using DnaSP v.6.12.03 [27]. Haplotype networks were constructed using PopArt [28] with the TCS algorithm [29] to estimate gene genealogy.

### 2.4. Ethical considerations

This study involved human participants (local volunteers) who collected adult mosquitoes using the human landing catch (HLC) and the animal-baited trap method in natural field settings. Participation was voluntary, and written informed consent was obtained from all collectors, with documentation witnessed accordingly. Ethical approval for these activities was granted by the Health Ethics Commission of the National Research and Innovation Agency (BRIN), under approval number 111/ KE.03/SK/05/2024.

## 3. Results

### 3.1. The Internal Transcribed Spacer 2 (ITS2) diversity, phylogeny, and polymorphism of *Anopheles tessellatus*

**Fig 2:**
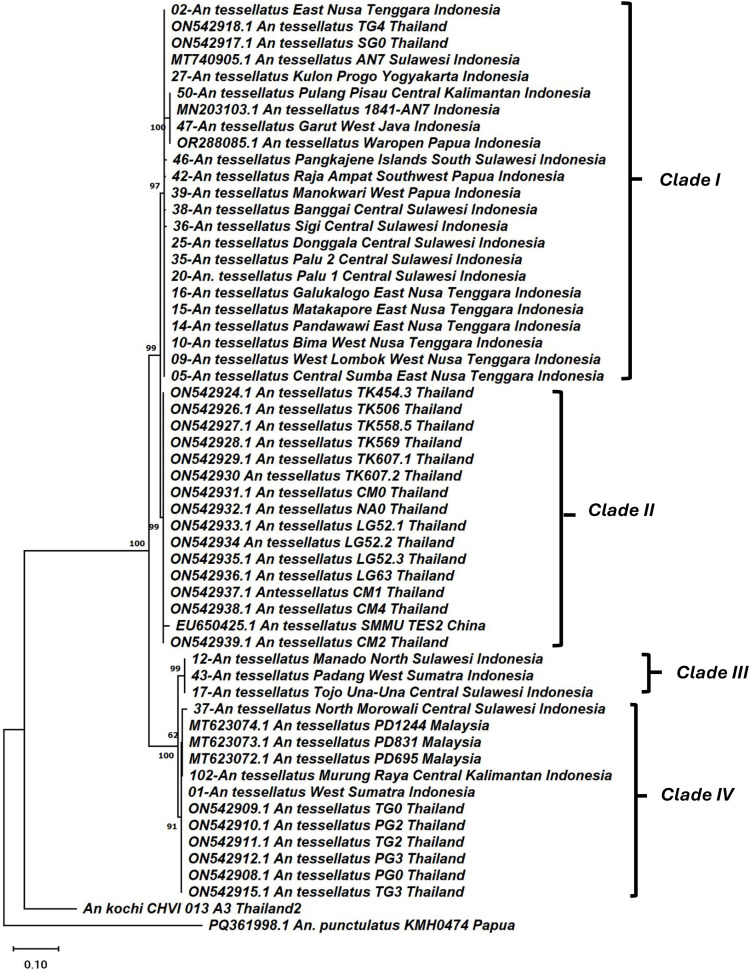
Phylogenetic analysis of the ITS2 sequences of the *Anopheles tessellatus* complex, rooted with *Anopheles punctulatus*. The phylogenetic tree was constructed using the Maximum Likelihood (ML) method with the Kimura 2-parameter evolutionary model in MEGA X, and bootstrap values were tested with 1,000 replicates.

Analysis of ITS2 rDNA sequences from 24 *An. tessellatus* samples collected at 23 locations across Indonesia, supplemented by 30 reference sequences from GenBank (23 sequences from Thailand, three sequences from Indonesia, three from Malaysia, and one from China), revealed the presence of four distinct *An. tessellatus* populations within Indonesia (Table 1, Figs 1, 2). The majority of study samples clustered within Clade I, encompassing specimens from Kulon Progo (Yogyakarta), Pulang Pisau (Central Kalimantan), Garut (West Java), Pangkajene Islands (South Sulawesi), Banggai, Sigi, Donggala, and Palu (Central Sulawesi), West Lombok and Bima (West Nusa Tenggara), Galukalogo, Matakapore, Pandawawi, and Central Sumba (East Nusa Tenggara), Raja Ampat (Southwest Papua), Waropen, and Manokwari (West Papua) (Accession numbers: PV929394-PV929396, PV929398-PV929400, PV929402-PV929406, PV929408-PV929410, PV929412-PV929414). Genetic distances within Clade I ranged from 0% to 0.13%, with the greatest divergence observed between the Garut sample and sequence from Clade II (the genetic distance within Clade II range from 0% to 0.95%), a clade predominantly composed of samples from Thailand and one from China, with no Indonesian samples (Fig 2; Tables 2, 3).

**Table 2:** Genetic distance of *Anopheles tessellatus* based on the ITS2 gene in Indonesia using p-distance.

*Anopheles tessellatus*	Seq01_West_Sumatra_Indonesia	Seq02_East_Nusa_Tenggara_Indonesia	Seq05_Central_Sumba_East_Nusa_Tenggara_Indonesia	Seq09_West_Lombok_West_Nusa_Tenggara_Indonesia	Seq10_Bima_West_Nusa_Tenggara_Indonesia	Seq12_Manado_North_Sulawesi_Indonesia	Seq14_Pandawawi_East_Nusa_Tenggara_Indonesia	Seq15_AMatakapore_East_Nusa_Tenggara_Indonesia	Seq16_Galukalogo_East_Nusa_Tenggara_Indonesia
Seq01_*An_tessellatus*_West_Sumatra_Indonesia									
Seq02_*An_tessellatus*_East_Nusa_Tenggara_Indonesia	0.08785								
Seq05_*An_tessellatus*_Central_Sumba_East_Nusa_Tenggara_Indonesia	0.08785	0.00000							
Seq09_*An_tessellatus*_West_Lombok_West_Nusa_Tenggara_Indonesia	0.08785	0.00000	0.00000						
Seq10_*An_tessellatus*_Bima_West_Nusa_Tenggara_Indonesia	0.08785	0.00000	0.00000	0.00000					
Seq12_*An_tessellatus*_Manado_North_Sulawesi_Indonesia	0.02026	0.10275	0.10275	0.10275	0.10275				
Seq14_*An_tessellatus*_Pandawawi_East_Nusa_Tenggara_Indonesia	0.08785	0.00000	0.00000	0.00000	0.00000	0.10275			
Seq15_*An_tessellatus*_Matakapore_East_Nusa_Tenggara_Indonesia	0.09056	0.00000	0.00000	0.00000	0.00000	0.10586	0.00000		
Seq16_*An_tessellatus*_Galukalogo_East_Nusa_Tenggara_Indonesia	0.08785	0.00000	0.00000	0.00000	0.00000	0.10275	0.00000	0.00000	
Seq17_*An_tessellatus*_Tojo_Una-Una_Central_Sulawesi_Indonesia	0.02026	0.10275	0.10275	0.10275	0.10275	0.00000	0.10275	0.10586	0.10275
Seq20_*An._tessellatus*_Palu_1_Central_Sulawesi_Indonesia	0.07317	0.00000	0.00000	0.00000	0.00000	0.07561	0.00000	0.00000	0.00000
Seq35_*An_tessellatus*_Palu_2_Central_Sulawesi_Indonesia	0.09524	0.00000	0.00000	0.00000	0.00000	0.11359	0.00000	0.00000	0.00000
Seq25_*An_tessellatus*_Donggala_Central_Sulawesi_Indonesia	0.08559	0.00000	0.00000	0.00000	0.00000	0.10573	0.00000	0.00000	0.00000
Seq36_*An_tessellatus*_Sigi_Central_Sulawesi_Indonesia	0.09478	0.00370	0.00370	0.00370	0.00370	0.11006	0.00370	0.00370	0.00370
Seq37_*An_tessellatus*_North_Morowali_Central_Sulawesi_Indonesia	0.01113	0.09981	0.09981	0.09981	0.09981	0.02783	0.09981	0.09634	0.09981
Seq38_*An_tessellatus*_Banggai_Central_Sulawesi_Indonesia	0.08745	0.00182	0.00182	0.00182	0.00182	0.10634	0.00182	0.00185	0.00182
Seq39_*An_tessellatus*_Manokwari_West_Papua_Indonesia	0.08818	0.00000	0.00000	0.00000	0.00000	0.10313	0.00000	0.00000	0.00000
Seq42_*An_tessellatus*_Raja_Ampat_Southwest_Papua_Indonesia	0.08972	0.00179	0.00179	0.00179	0.00179	0.10092	0.00179	0.00185	0.00179
Swq43_*An_tessellatus*_Padang_West_Sumatra_Indonesia	0.02037	0.10332	0.10332	0.10332	0.10332	0.00000	0.10332	0.10646	0.10332
Seq46_*An_tessellatus*_Pangkajene_Islands_South_Sulawesi_Indonesia	0.08824	0.00432	0.00432	0.00432	0.00432	0.10841	0.00432	0.00432	0.00432
Seq47_*An_tessellatus*_Garut_West_Java_Indonesia	0.09925	0.01077	0.01077	0.01077	0.01077	0.11397	0.01077	0.00000	0.01077
Seq50_*An_tessellatus*_Pulang_Pisau_Central_Kalimantan_Indonesia	0.09925	0.01077	0.01077	0.01077	0.01077	0.11397	0.01077	0.00000	0.01077
Seq27_*An_tessellatus*_Kulon_Progo_Yogyakarta_Indonesia	0.06548	0.00000	0.00000	0.00000	0.00000	0.06845	0.00000	0.00000	0.00000
Seq102_*An_tessellatus*_Murung_Raya_Central_Kalimantan_Indonesia	0.00184	0.08972	0.08972	0.08972	0.08972	0.01842	0.08972	0.09249	0.08972
EU650425.1_*An_tessellatus*_SMMU_TES2_China	0.10078	0.02788	0.02788	0.02788	0.02788	0.11597	0.02788	0.02788	0.02788
MN203103.1_*An_tessellatus*_1841-AN7_Indonesia	0.10093	0.01254	0.01254	0.01254	0.01254	0.11560	0.01254	0.00000	0.01254
MT623072.1_*An_tessellatus*_PD695_Malaysia	0.00185	0.09006	0.09006	0.09006	0.09006	0.01848	0.09006	0.09249	0.09006
MT623073.1_*An_tessellatus*_PD831_Malaysia	0.00185	0.09006	0.09006	0.09006	0.09006	0.01848	0.09006	0.09249	0.09006
MT623074.1_*An_tessellatus*_PD1244_Malaysia	0.00185	0.09006	0.09006	0.09006	0.09006	0.01848	0.09006	0.09249	0.09006
MT740905.1_*An_tessellatus*_AN7_Sulawesi_Indonesia	0.09162	0.00000	0.00000	0.00000	0.00000	0.10707	0.00000	0.00000	0.00000
OR288085.1_*An_tessellatus*_Waropen_Papua_Indonesia	0.09925	0.01077	0.01077	0.01077	0.01077	0.11397	0.01077	0.00000	0.01077
ON542908.1_*An_tessellatus*_PG0_Thailand	0.00000	0.09162	0.09162	0.09162	0.09162	0.02111	0.09162	0.09162	0.09162
ON542909.1_*An_tessellatus*_TG0_Thailand	0.00000	0.09162	0.09162	0.09162	0.09162	0.02111	0.09162	0.09162	0.09162
ON542910.1_*An_tessellatus*_PG2_Thailand	0.00000	0.09198	0.09198	0.09198	0.09198	0.02119	0.09198	0.09198	0.09198
ON542911.1_*An_tessellatus*_TG2_Thailand	0.00000	0.09198	0.09198	0.09198	0.09198	0.02119	0.09198	0.09198	0.09198
ON542912.1_*An_tessellatus*_PG3_Thailand	0.00000	0.09592	0.09592	0.09592	0.09592	0.02209	0.09592	0.09592	0.09592
ON542915.1_*An_tessellatus*_TG3_Thailand	0.00000	0.09252	0.09252	0.09252	0.09252	0.02132	0.09252	0.09252	0.09252
*Anopheles tessellatus*	Seq17_Tojo_Una-Una_Central_Sulawesi_Indonesia	Seq20_APalu_1_Central_Sulawesi_Indonesia	Seq46_Pangkajene_Islands_South_Sulawesi_Indonesia	Seq25_Donggala_Central_Sulawesi_Indonesia	Seq36_Sigi_Central_Sulawesi_Indonesia	Seq37_North_Morowali_Central_Sulawesi_Indonesia	Seq38_Banggai_Central_Sulawesi_Indonesia	Seq39_Manokwari_West_Papua_Indonesia	Seq42_Raja_Ampat_Southwest_Papua_Indonesia
ON542917.1_*An_tessellatus*_SG0_Thailand	0.10707	0.00000	0.00000	0.00000	0.00373	0.09747	0.00187	0.00000	0.00187
ON542918.1_*An_tessellatus*_TG4_Thailand	0.11200	0.00000	0.00000	0.00000	0.00390	0.10204	0.00195	0.00000	0.00195
ON542924.1_*An_tessellatus*_TK454.3_Thailand	0.10365	0.01675	0.01590	0.01515	0.01876	0.09393	0.01689	0.01507	0.01689
ON542926.1_*An_tessellatus*_TK506_Thailand	0.10365	0.01675	0.01590	0.01515	0.01876	0.09393	0.01689	0.01507	0.01689
ON542927.1_*An_tessellatus*_TK558.5_Thailand	0.10365	0.01675	0.01590	0.01515	0.01876	0.09393	0.01689	0.01507	0.01689
ON542928.1_*An_tessellatus*_TK569_Thailand	0.10365	0.01675	0.01590	0.01515	0.01876	0.09393	0.01689	0.01507	0.01689
ON542929.1_*An_tessellatus*_TK607.1_Thailand	0.10365	0.01675	0.01590	0.01515	0.01876	0.09393	0.01689	0.01507	0.01689
ON542930_*An_tessellatus*_TK607.2_Thailand	0.10365	0.01675	0.01590	0.01515	0.01876	0.09393	0.01689	0.01507	0.01689
ON542931.1_*An_tessellatus*_CM0_Thailand	0.10365	0.01675	0.01590	0.01515	0.01876	0.09393	0.01689	0.01507	0.01689
ON542932.1_*An_tessellatus*_NA0_Thailand	0.10365	0.01675	0.01590	0.01515	0.01876	0.09393	0.01689	0.01507	0.01689
ON542933.1_*An_tessellatus*_LG52.1_Thailand	0.10365	0.01675	0.01590	0.01515	0.01876	0.09393	0.01689	0.01507	0.01689
ON542934_*An_tessellatus*_LG52.2_Thailand	0.10365	0.01675	0.01590	0.01515	0.01876	0.09393	0.01689	0.01507	0.01689
ON542935.1_*An_tessellatus*_LG52.3_Thailand	0.10365	0.01675	0.01590	0.01515	0.01876	0.09393	0.01689	0.01507	0.01689
ON542936.1_*An_tessellatus*_LG63_Thailand	0.10365	0.01675	0.01590	0.01515	0.01876	0.09393	0.01689	0.01507	0.01689
ON542937.1_*Antessellatus*_CM1_Thailand	0.10526	0.01707	0.01616	0.01542	0.01905	0.09543	0.01714	0.01524	0.01714
ON542938.1_*An_tessellatus*_CM4_Thailand	0.10526	0.01707	0.01616	0.01542	0.01905	0.09543	0.01714	0.01524	0.01714
ON542939.1_*An_tessellatus*_CM2_Thailand	0.10445	0.01691	0.01603	0.01528	0.01890	0.09467	0.01701	0.01512	0.01701
*Anopheles tessellatus*	Seq43_Padang_West_Sumatra_Indonesia	Seq46_Pangkajene_Islands_South_Sulawesi_Indonesia	Seq47_Garut_West_Java_Indonesia	Seq50_Pulang_Pisau_Central_Kalimantan_Indonesia	Seq27_Kulon_Progo_Yogyakarta_Indonesia	Seq102_Murung_Raya_Central_Kalimantan_Indonesia	EU650425.1_SMMU_TES2_China	MN203103.1_1841-AN7_Indonesia	MT623072.1_PD695_Malaysia
Seq46_*An_tessellatus*_Pangkajene_Islands_South_Sulawesi_Indonesia	0.10841								
Seq47_*An_tessellatus*_Garut_West_Java_Indonesia	0.11460	0.00432							
Seq50_*An_tessellatus*_Pulang_Pisau_Central_Kalimantan_Indonesia	0.11460	0.00432	0.00000						
Seq27_*An_tessellatus*_Kulon_Progo_Yogyakarta_Indonesia	0.06907	0.00000	0.00000	0.00000					
Seq102_*An_tessellatus*_Murung_Raya_Central_Kalimantan_Indonesia	0.01852	0.09050	0.10112	0.10112	0.06845				
EU650425.1_*An_tessellatus*_SMMU_TES2_China	0.11663	0.01739	0.02788	0.02788	0.00581	0.10271			
MN203103.1_*An_tessellatus*_1841-AN7_Indonesia	0.11624	0.00432	0.00000	0.00000	0.00000	0.10280	0.02783		
MT623072.1_*An_tessellatus*_PD695_Malaysia	0.01859	0.09050	0.09944	0.09944	0.06845	0.00000	0.10251	0.09925	
MT623073.1_*An_tessellatus*_PD831_Malaysia	0.01859	0.09050	0.09944	0.09944	0.06845	0.00000	0.10251	0.09925	0.00000
MT623074.1_*An_tessellatus*_PD1244_Malaysia	0.01859	0.09050	0.09944	0.09944	0.06845	0.00000	0.10251	0.09925	0.00000
MT740905.1_*An_tessellatus*_AN7_Sulawesi_Indonesia	0.10769	0.00432	0.00000	0.00000	0.00000	0.09357	0.02809	0.00000	0.09339
OR288085.1_*An_tessellatus*_Waropen_Papua_Indonesia	0.11460	0.00432	0.00000	0.00000	0.00000	0.10112	0.02783	0.00000	0.09925
ON542908.1_*An_tessellatus*_PG0_Thailand	0.02124	0.08824	0.09162	0.09162	0.06548	0.00192	0.10156	0.09144	0.00192
ON542909.1_*An_tessellatus*_TG0_Thailand	0.02124	0.08824	0.09162	0.09162	0.06548	0.00192	0.10156	0.09144	0.00192
ON542910.1_*An_tessellatus*_PG2_Thailand	0.02124	0.08824	0.09198	0.09198	0.06587	0.00193	0.10196	0.09180	0.00192
ON542911.1_*An_tessellatus*_TG2_Thailand	0.02124	0.08824	0.09198	0.09198	0.06587	0.00193	0.10196	0.09180	0.00192
ON542912.1_*An_tessellatus*_PG3_Thailand	0.02209	0.09242	0.09592	0.09592	0.07029	0.00201	0.10656	0.09592	0.00201
ON542915.1_*An_tessellatus*_TG3_Thailand	0.02132	0.08864	0.09252	0.09252	0.06647	0.00194	0.10256	0.09234	0.00193
ON542917.1_*An_tessellatus*_SG0_Thailand	0.10769	0.00432	0.00000	0.00000	0.00000	0.09357	0.02809	0.00000	0.09339
ON542918.1_*An_tessellatus*_TG4_Thailand	0.11200	0.00451	0.00000	0.00000	0.00000	0.09796	0.02941	0.00000	0.09796
ON542924.1_*An_tessellatus*_TK454.3_Thailand	0.10425	0.01739	0.01501	0.01501	0.00581	0.09002	0.01311	0.01498	0.08984
ON542926.1_*An_tessellatus*_TK506_Thailand	0.10425	0.01739	0.01501	0.01501	0.00581	0.09002	0.01311	0.01498	0.08984
ON542927.1_*An_tessellatus*_TK558.5_Thailand	0.10425	0.01739	0.01501	0.01501	0.00581	0.09002	0.01311	0.01498	0.08984
ON542928.1_*An_tessellatus*_TK569_Thailand	0.10425	0.01739	0.01501	0.01501	0.00581	0.09002	0.01311	0.01498	0.08984
ON542929.1_*An_tessellatus*_TK607.1_Thailand	0.10425	0.01739	0.01501	0.01501	0.00581	0.09002	0.01311	0.01498	0.08984
ON542930.1_*An_tessellatus*_TK607.2_Thailand	0.10425	0.01739	0.01501	0.01501	0.00581	0.09002	0.01311	0.01498	0.08984
ON542931.1_*An_tessellatus*_CM0_Thailand	0.10425	0.01739	0.01501	0.01501	0.00581	0.09002	0.01311	0.01498	0.08984
ON542932.1_*An_tessellatus*_NA0_Thailand	0.10425	0.01739	0.01501	0.01501	0.00581	0.09002	0.01311	0.01498	0.08984
ON542933.1_*An_tessellatus*_LG52.1_Thailand	0.10425	0.01739	0.01501	0.01501	0.00581	0.09002	0.01311	0.01498	0.08984
ON542934.1_*An_tessellatus*_LG52.2_Thailand	0.10425	0.01739	0.01501	0.01501	0.00581	0.09002	0.01311	0.01498	0.08984
ON542935.1_*An_tessellatus*_LG52.3_Thailand	0.10425	0.01739	0.01501	0.01501	0.00581	0.09002	0.01311	0.01498	0.08984
ON542936.1_*An_tessellatus*_LG63_Thailand	0.10425	0.01739	0.01501	0.01501	0.00581	0.09002	0.01311	0.01498	0.08984
ON542937.1_*An_tessellatus*_CM1_Thailand	0.10526	0.01758	0.01524	0.01524	0.00595	0.09145	0.01331	0.01521	0.09127
ON542938.1_*An_tessellatus*_CM4_Thailand	0.10526	0.01758	0.01524	0.01524	0.00595	0.09145	0.01331	0.01521	0.09127
ON542939.1_*An_tessellatus*_CM2_Thailand	0.10445	0.01743	0.01512	0.01512	0.00588	0.09073	0.01321	0.01509	0.09055
*Anopheles tessellatus*	MT62307 3.1_PD83 1_Malaysia	MT6230 74.1_P D1244_ Malaysia	MT7409 05.1_A N7_Sula wesi_Indonesia	OR28808 5.1_Waro pen_Papu a_Indones ia	ON5429 08.1_P G0_Thailand	ON5429 09.1_T G0_Thailand	ON5429 10.1_P G2_Thailand	ON5429 11.1_T G2_Thailand	ON54291 2.1_PG3_ Thailand
MT623074.1_*An_tessellatus*_PD1244_Malaysia	0.00000								
MT740905.1_*An_tessellatus*_AN7_Sulawesi_Indonesia	0.09339	0.09339							
OR288085.1_*An_tessellatus*_Waropen_Papua_Indonesia	0.09925	0.09925	0.00000						
ON542908.1_*An_tessellatus*_PG0_Thailand	0.00192	0.00192	0.09144	0.09144					
ON542909.1_*An_tessellatus*_TG0_Thailand	0.00192	0.00192	0.09144	0.09144	0.00000				
ON542910.1_*An_tessellatus*_PG2_Thailand	0.00192	0.00192	0.09180	0.09180	0.00000	0.00000			
ON542911.1_*An_tessellatus*_TG2_Thailand	0.00192	0.00192	0.09180	0.09180	0.00000	0.00000	0.00000		
ON542912.1_*An_tessellatus*_PG3_Thailand	0.00201	0.00201	0.09592	0.09592	0.00000	0.00000	0.00000	0.00000	
ON542915.1_*An_tessellatus*_TG3_Thailand	0.00193	0.00193	0.09234	0.09234	0.00000	0.00000	0.00000	0.00000	0.00000
ON542917.1_*An_tessellatus*_SG0_Thailand	0.09339	0.09339	0.00000	0.00000	0.09144	0.09144	0.09180	0.09180	0.09592
ON542918.1_*An_tessellatus*_TG4_Thailand	0.09796	0.09796	0.00000	0.00000	0.09592	0.09592	0.09592	0.09592	0.09592
ON542924.1_*An_tessellatus*_TK454.3_Thailand	0.08984	0.08984	0.01498	0.01498	0.08789	0.08789	0.08824	0.08824	0.09221
ON542926.1_*An_tessellatus*_TK506_Thailand	0.08984	0.08984	0.01498	0.01498	0.08789	0.08789	0.08824	0.08824	0.09221
ON542927.1_*An_tessellatus*_TK558.5_Thailand	0.08984	0.08984	0.01498	0.01498	0.08789	0.08789	0.08824	0.08824	0.09221
ON542928.1_*An_tessellatus*_TK569_Thailand	0.08984	0.08984	0.01498	0.01498	0.08789	0.08789	0.08824	0.08824	0.09221
ON542929.1_*An_tessellatus*_TK607.1_Thailand	0.08984	0.08984	0.01498	0.01498	0.08789	0.08789	0.08824	0.08824	0.09221
ON542930.1_*An_tessellatus*_TK607.2_Thailand	0.08984	0.08984	0.01498	0.01498	0.08789	0.08789	0.08824	0.08824	0.09221
ON542931.1_*An_tessellatus*_CM0_Thailand	0.08984	0.08984	0.01498	0.01498	0.08789	0.08789	0.08824	0.08824	0.09221
ON542932.1_*An_tessellatus*_NA0_Thailand	0.08984	0.08984	0.01498	0.01498	0.08789	0.08789	0.08824	0.08824	0.09221
ON542933.1_*An_tessellatus*_LG52.1_Thailand	0.08984	0.08984	0.01498	0.01498	0.08789	0.08789	0.08824	0.08824	0.09221
ON542934.1_*An_tessellatus*_LG52.2_Thailand	0.08984	0.08984	0.01498	0.01498	0.08789	0.08789	0.08824	0.08824	0.09221
ON542935.1_*An_tessellatus*_LG52.3_Thailand	0.08984	0.08984	0.01498	0.01498	0.08789	0.08789	0.08824	0.08824	0.09221
ON542936.1_*An_tessellatus*_LG63_Thailand	0.08984	0.08984	0.01498	0.01498	0.08789	0.08789	0.08824	0.08824	0.09221
ON542937.1_*An_tessellatus*_CM1_Thailand	0.09127	0.09127	0.01521	0.01521	0.08929	0.08929	0.08929	0.08929	0.09221
ON542938.1_*An_tessellatus*_CM4_Thailand	0.09127	0.09127	0.01521	0.01521	0.08929	0.08929	0.08929	0.08929	0.09221
ON542939.1_*An_tessellatus*_CM2_Thailand	0.09055	0.09055	0.01509	0.01509	0.08858	0.08858	0.08858	0.08858	0.09221
*Anopheles tessellatus*	ON5429 15.1_TG 3_Thaila nd	ON542 917.1_ SG0_Thailand	ON542 918.1_ TG4_Thailand	ON5429 24.1_TK 454.3_Thailand	ON542 926.1_ TK506 _Thaila nd	ON542 927.1_ TK558. 5_Thailand	ON542 928.1_ TK569 _Thaila nd	ON542 929.1_ TK607. 1_Thailand	ON5429 30_TK60 7.2_Thailand
ON542917.1_*An_tessellatus*_SG0_Thailand	0.09234								
ON542918.1_*An_tessellatus*_TG4_Thailand	0.09592	0.00000							
ON542924.1_*An_tessellatus*_TK454.3_Thailand	0.08876	0.01498	0.01569						
ON542926.1_*An_tessellatus*_TK506_Thailand	0.08876	0.01498	0.01569	0.00000					
ON542927.1_*An_tessellatus*_TK558.5_Thailand	0.08876	0.01498	0.01569	0.00000	0.00000				
ON542928.1_*An_tessellatus*_TK569_Thailand	0.08876	0.01498	0.01569	0.00000	0.00000	0.00000			
ON542929.1_*An_tessellatus*_TK607.1_Thailand	0.08876	0.01498	0.01569	0.00000	0.00000	0.00000	0.00000		
ON542930.1_*An_tessellatus*_TK607.2_Thailand	0.08876	0.01498	0.01569	0.00000	0.00000	0.00000	0.00000	0.00000	
ON542931.1_*An_tessellatus*_CM0_Thailand	0.08876	0.01498	0.01569	0.00000	0.00000	0.00000	0.00000	0.00000	0.00000
ON542932.1_*An_tessellatus*_NA0_Thailand	0.08876	0.01498	0.01569	0.00000	0.00000	0.00000	0.00000	0.00000	0.00000
ON542933.1_*An_tessellatus*_LG52.1_Thailand	0.08876	0.01498	0.01569	0.00000	0.00000	0.00000	0.00000	0.00000	0.00000
ON542934.1_*An_tessellatus*_LG52.2_Thailand	0.08876	0.01498	0.01569	0.00000	0.00000	0.00000	0.00000	0.00000	0.00000
ON542935.1_*An_tessellatus*_LG52.3_Thailand	0.08876	0.01498	0.01569	0.00000	0.00000	0.00000	0.00000	0.00000	0.00000
ON542936.1_*An_tessellatus*_LG63_Thailand	0.08876	0.01498	0.01569	0.00000	0.00000	0.00000	0.00000	0.00000	0.00000
ON542937.1_*An_tessellatus*_CM1_Thailand	0.08929	0.01521	0.01569	0.00000	0.00000	0.00000	0.00000	0.00000	0.00000
ON542938.1_*An_tessellatus*_CM4_Thailand	0.08929	0.01521	0.01569	0.00000	0.00000	0.00000	0.00000	0.00000	0.00000
ON542939.1_*An_tessellatus*_CM2_Thailand	0.08876	0.01509	0.01569	0.00000	0.00000	0.00000	0.00000	0.00000	0.00000
*Anopheles tessellatus*	ON5429 31.1_CM 0_Thaila nd	ON542 932.1_ NA0_Thailand	ON542 933.1_ LG52.1 _Thaila nd	ON5429 34_LG52 .2_Thaila nd	ON542 935.1_ LG52.3 _Thaila nd	ON542 936.1_ LG63_ Thailand	ON542 937.1_ CM1_Thailand	ON542 938.1_ CM4_Thailand	ON5429 39.1_CM 2_Thaila nd
ON542931.1_*An_tessellatus*_CM0_Thailand									
ON542932.1_*An_tessellatus*_NA0_Thailand	0.00000								
ON542933.1_*An_tessellatus*_LG52.1_Thailand	0.00000	0.00000							
ON542934.1_*An_tessellatus*_LG52.2_Thailand	0.00000	0.00000	0.00000						
ON542935.1_*An_tessellatus*_LG52.3_Thailand	0.00000	0.00000	0.00000	0.00000					
ON542936.1_*An_tessellatus*_LG63_Thailand	0.00000	0.00000	0.00000	0.00000	0.00000				
ON542937.1_*An_tessellatus*_CM1_Thailand	0.00000	0.00000	0.00000	0.00000	0.00000	0.00000			
ON542938.1_*An_tessellatus*_CM4_Thailand	0.00000	0.00000	0.00000	0.00000	0.00000	0.00000	0.00000		
ON542939.1_*An_tessellatus*_CM2_Thailand	0.00000	0.00000	0.00000	0.00000	0.00000	0.00000	0.00000	0.00000	

**Table 3:** Mean genetic distances within and between ITS2 clades of *Anopheles tessellatus* estimated using the Kimura 2-parameter model.

Clade	1	2	3	4
**1**	**0.0023 ± 0.0007**	0.0054	0.0154	0.0152
**2**		**0.0017 ± 0.0006**	0.0153	0.0148
**3**			**0.0142 ± 0.0038**	0.0047
**4**				**0.0010 ± 0.0010**

**Note:** Diagonal values indicate within-clade mean genetic distances (mean ± SE); upper-triangular values indicate between-clade mean genetic distances.

Clade III, notably distinct, comprised samples from geographically distant locations: Padang (West Sumatra), Tojo Una-Una (Central Sulawesi), and Manado (North Sulawesi) (PV929396, PV929401, PV929411). Intra-clade genetic distances ranged from 0.14% to 1.14%, with the Padang samples exhibiting the highest divergence. Clade IV included samples from the South Coast of West Sumatra, North Morowali (Central Sulawesi), and Murung Raya (Central Kalimantan) (Accession numbers: PV929392, PV929407, PV929414), with genetic distances within this group ranging between 0.05% and 0.09% (Fig 2; Tables 2, 3).

Despite the presence of four genetically distinct populations, inter-clade genetic distances ranged from 0.01% to 1.03%. Clade I demonstrated the greatest diversity and the broadest geographic distribution across Indonesia. Clade II appears to represent a population widely distributed in Thailand and China. Samples assigned to Clade III correspond to the Sulawesi lineage, currently identified only on the island of Sulawesi. Clades I and III represent unique populations endemic to Indonesia. Conversely, Clade IV displays a wider distribution across three Southeast Asian countries, including Malaysia, Indonesia, and Thailand (Fig 2; Tables 2, 3; S1 Fig).

**Fig 3:**
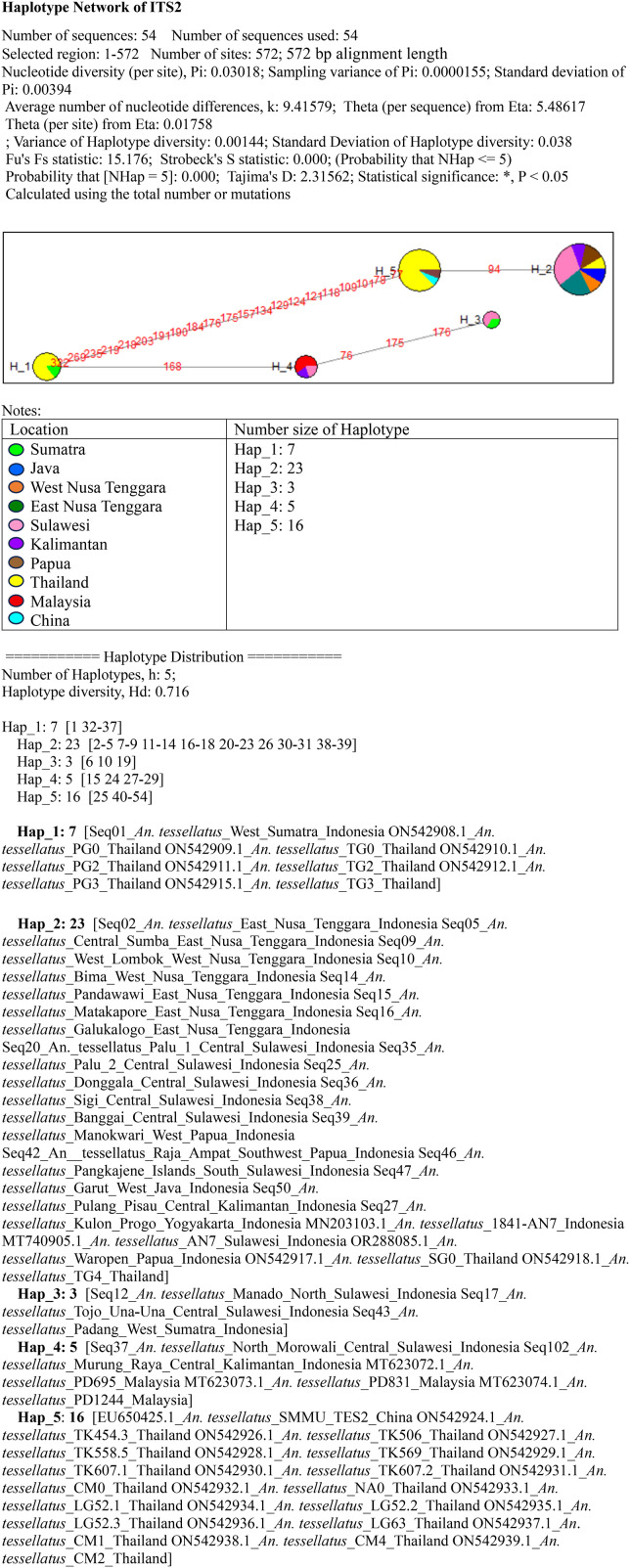
Haplotype network of *Anopheles tessellatus* based on ITS2 sequences. The Fig illustrates the frequency of each ITS2 haplotype across study sites, with connections representing the number of mutations separating haplotypes and their geographic distribution.

**Fig 4:**
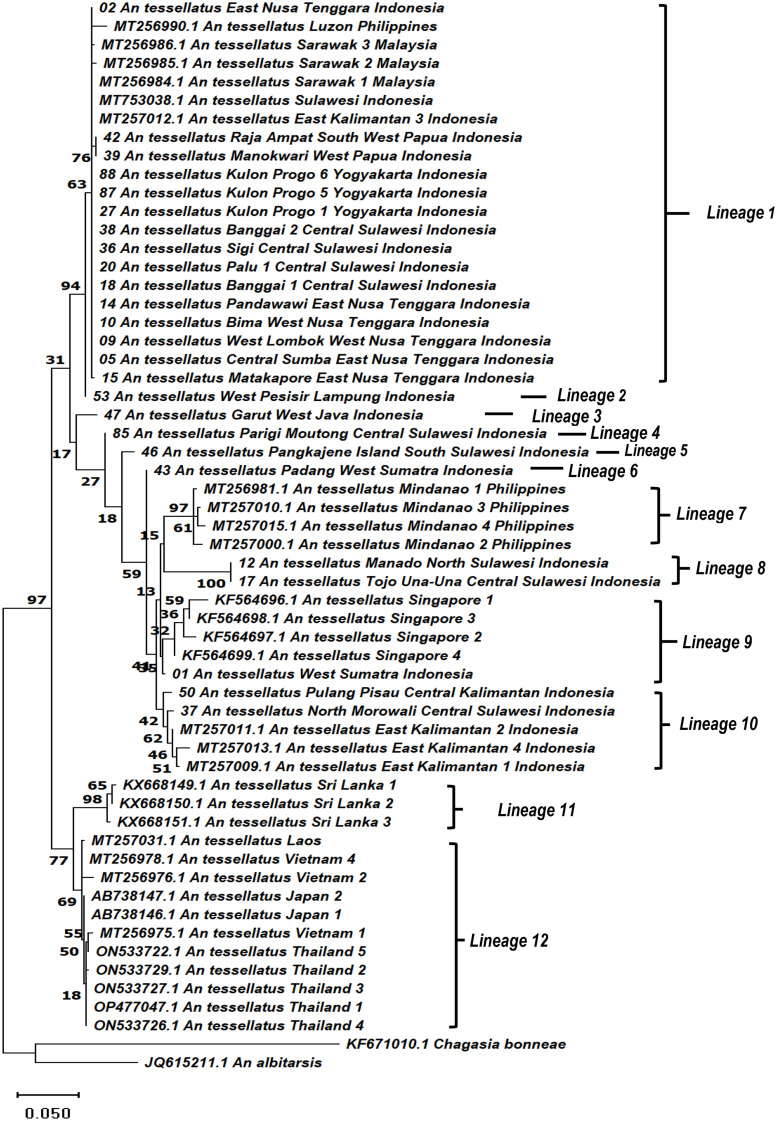
Phylogenetic analysis of COI sequences of *Anopheles tessellatus*, rooted with *Chagasia bonneae* as the outgroup. The phylogenetic tree was constructed using the General Time Reversible (GTR+I) model in MEGA X, with bootstrap support estimated from 1,000 replicates to assess tree reliability.

ITS2 haplotype analysis reveals distinct genetic diversity patterns in *An. tessellatus* populations across Indonesia and reference sequences from other regions in Asia (Figs 3, 4). Five distinct haplotypes were identified from 54 sequences, supported by two variable sites, with a haplotype diversity (Hd) of 0.716, indicating moderate intraspecific variation suitable for population studies in vector mosquitoes [30]. Hap1, the most frequent (n=7), includes sequences from West Sumatra (Indonesia) and Thailand, differing at positions 32-37, suggesting a widespread ancestral lineage. Hap2 (n=23) dominates Indonesian samples from East Nusa Tenggara, West Nusa Tenggara, Central Sulawesi, West Papua, South Sulawesi, West Java, Central Kalimantan, and Yogyakarta, with mutations at positions 2-5, 7-9, 11-14, 16-18, 20-23, 26, 30-31, and 38-39, reflecting regional diversification. Hap3 (n=3) appears limited to North and Central Sulawesi (Manado, Tojo Una-Una), and West Sumatra, varying at sites 6, 10, and 19. Hap4 (n=5) links Central Sulawesi, Central Kalimantan, and Malaysia (positions 15, 24, 27-29). Hap5 (n=16), primarily Thai sequences with one Chinese reference, shows extensive variation at sites 25 and 40-54, highlighting geographic structuring. Haplotype networks, typically constructed via median-joining or TCS methods, visualize mutational steps between haplotypes, with Hap1 as a central node linking Indonesian-Thai populations and radiating to Hap2 (most diverged, multi-site changes) and minor haplotypes (Hap3-5). The presence of short mutational branches among Indonesian Hap2 sequences indicate recent population expansion or gene flow across islands, while longer branches to Hap5 suggest historical isolation or concerted evolution minimizing ITS2 intraspecific polymorphism. This structure implies that *An. tessellatus* comprises cryptic lineages, with Hap2 potentially adapted to diverse Indonesian ecologies, warranting vector competence assessments for malaria control (Fig 3).

### 3.2. The Cytochrome Oxidase I gene (COI) diversity, phylogeny, and polymorphism of *Anopheles tessellatus*

Analysis of COI sequences to identify maternal lineages revealed that all 25 samples from this study (22 locations across Indonesia), together with 31 reference sequences from GenBank (five from Indonesia, three from Malaysia, four from Singapore, five from Philippines, five from Thailand, three from Sri Lanka, two from Japan, three from Vietnam, one from Laos) grouped into 12 distinct lineages within the *An. tessellatus* populations. Lineage 1 comprised samples from Kulon Progo (Yogyakarta) (PX 363429), Banggai, Sigi, and Palu (Central Sulawesi (PV960007, PV960009, PV960008, PV960011), Pandawawi, Central Sumba, and Matakapore (East Nusa Tenggara) (PV960004, PV960000, PV960005), Bima and West Lombok (West Nusa Tenggara) (PV960002, PV960001), Raja Ampat and Manokwari (West Papua) (PV960017, PX106897), along with six samples of GenBank reference sequences from East Kalimantan (MT257012), Sulawesi (MT753038), Sarawak (MT256986, MT256985, MT256984), and the Philippines (MT256990) (Table 1, Fig 4).

Lineage 2 was represented solely by specimens from West Pesisir, Lampung (PV960018), while lineage 3 contained only sample from Garut (West Java) (PX106893). Subsequently, lineages 4, 5, and 6, each contained samples from Parigi Moutong (Central Sulawesi) (PX106895), Pangkajene Islands (South Sulawesi) (PX106896), and Padang (West Sumatra) (PX106894), respectively. Lineage 7 consisted exclusively of GenBank references from the Philippines (MT256981, MT257000, MT257010, MT257015). Lineage 8 included specimens from Manado (North Sulawesi) (PV960003) and Tojo Una-Una (Central Sulawesi) (PV960006). Lineage 9 encompassed samples from the South Coast of West Sumatra (PV936689) and four GenBank references from Singapore (KF564696-KF564699). Lineage 10 contained specimens solely from Indonesia, including Pulang Pisau (Central Kalimantan) (PX107476), GenBank references from East Kalimantan (MT257009, MT257011, MT257013), and North Morowali (Central Sulawesi) (PV960010). Lineage 11 was represented only by three GenBank samples from Sri Lanka (KX668149-KX668151), whereas lineage 12 comprised a mixture of GenBank sequences from four Asian countries: Thailand (ON533722, ON533726, ON533727, ON533729, OP477047), Japan (AB738146-AB738147), Vietnam (MT256975-MT256976, MT256978), and Laos (MT257031). Phylogenetic rooting was achieved using *Chagasia bonneae* (KF671010) and *Anopheles albitarsis* (JQ615211) as outgroups (Fig 4).

The majority of *An. tessellatus* samples in this study fell within Lineage 1, exhibiting minimal genetic divergence with a distance ranging from 0% to 0.04%. Apart from Lineage 1, Indonesian specimens also clustered in lineages 2, 3, 4, 5, 6, 8, 9, and 10. Notably, samples had genetic distances ranging from 0.04% to 0.6%. Inter-lineage COI sequence divergences ranged between 0.03% and 0.6%. These findings indicate that despite geographic separation across various regions and islands in Indonesia over extended evolutionary timescales, specifically since at least the Triassic Period of the Mesozoic Era, approximately 2 million years ago [31], *An. tessellatus* populations in Indonesia remain part of a single maternal lineage (Fig 4, Tables 4, 5; S2 Fig).

**Table 4:** Genetic distance of *Anopheles tessellatus* based on the COI gene in Indonesia using p-distance.

*Anopheles tessellatus*	01_West_Sumatra_Indonesia	02_East_Nusa_Tenggara_Indonesia	05_Central_Sumba_East_Nusa_Tenggara_Indonesia	09_West_Lombok_West_Nusa_Tenggara_Indonesia	10_Bima_West_Nusa_Tenggara_Indonesia	12_Manado_North_Sulawesi_Indonesia	14_Pandawawi_East_Nusa_Tenggara_Indonesia	15_Matakapore_East_Nusa_Tenggara_Indonesia	17_Tojo_Una-Una_Central_Sulawesi_Indonesia	
01_*An_tessellatus*_West_Sumatra_Indonesia										
02_*An_tessellatus*_East_Nusa_Tenggara_Indonesia	0.05975									
05_*An_tessellatus*_Central_Sumba_East_Nusa_Tenggara_Indonesia	0.05975	0.00000								
09_*An_tessellatus*_West_Lombok_West_Nusa_Tenggara_Indonesia	0.05975	0.00000	0.00000							
10_*An_tessellatus*_Bima_West_Nusa_Tenggara_Indonesia	0.05975	0.00000	0.00000	0.00000						
12_*An_tessellatus*_Manado_North_Sulawesi_Indonesia	0.04245	0.08333	0.08333	0.08333	0.08333					
14_*An_tessellatus*_Pandawawi_East_Nusa_Tenggara_Indonesia	0.05975	0.00000	0.00000	0.00000	0.00000	0.08333				
15_*An_tessellatus*_Matakapore_East_Nusa_Tenggara_Indonesia	0.05818	0.00157	0.00157	0.00157	0.00157	0.08176	0.00157			
17_*An_tessellatus*_Tojo_Una-Una_Central_Sulawesi_Indonesia	0.04245	0.08333	0.08333	0.08333	0.08333	0.00000	0.08333	0.08176		
18_*An_tessellatus*_Banggai_1_Central_Sulawesi_Indonesia	0.05975	0.00000	0.00000	0.00000	0.00000	0.08333	0.00000	0.00157	0.08333	
20_*An_tessellatus*_Palu_1_Central_Sulawesi_Indonesia	0.05975	0.00000	0.00000	0.00000	0.00000	0.08333	0.00000	0.00157	0.08333	
36_*An_tessellatus*_Sigi_Central_Sulawesi_Indonesia	0.05975	0.00000	0.00000	0.00000	0.00000	0.08333	0.00000	0.00157	0.08333	
37_*An_tessellatus*_North_Morowali_Central_Sulawesi_Indonesia	0.01730	0.05818	0.05818	0.05818	0.05818	0.04245	0.05818	0.05660	0.04245	
38_*An_tessellatus*_Banggai_2_Central_Sulawesi_Indonesia	0.05975	0.00000	0.00000	0.00000	0.00000	0.08333	0.00000	0.00157	0.08333	
27_*An_tessellatus*_Kulon_Progo_1_Yogyakarta_Indonesia	0.05975	0.00000	0.00000	0.00000	0.00000	0.08333	0.00000	0.00157	0.08333	
87_*An_tessellatus*_Kulon_Progo_5_Yogyakarta_Indonesia	0.05975	0.00000	0.00000	0.00000	0.00000	0.08333	0.00000	0.00157	0.08333	
88_*An_tessellatus*_Kulon_Progo_6_Yogyakarta_Indonesia	0.05975	0.00000	0.00000	0.00000	0.00000	0.08333	0.00000	0.00157	0.08333	
42_*An_tessellatus*_Raja_Ampat_South_West_Papua_Indonesia	0.05818	0.00314	0.00314	0.00314	0.00314	0.08176	0.00314	0.00472	0.08176	
39_*An_tessellatus*_Manokwari_West_Papua_Indonesia	0.05818	0.00314	0.00314	0.00314	0.00314	0.08176	0.00314	0.00472	0.08176	
47_*An_tessellatus*_Garut_West_Java_Indonesia	0.03931	0.03145	0.03145	0.03145	0.03145	0.05189	0.03145	0.02987	0.05189	
43_*An_tessellatus*_Padang_West_Sumatra_Indonesia	0.01258	0.05189	0.05189	0.05189	0.05189	0.04874	0.05189	0.05031	0.04874	
85_*An_tessellatus*_Parigi_Moutong_Central_Sulawesi_Indonesia	0.03616	0.03459	0.03459	0.03459	0.03459	0.05660	0.03459	0.03302	0.05660	
46_*An_tessellatus*_Pangkajene_Island_South_Sulawesi_Indonesia	0.03302	0.03774	0.03774	0.03774	0.03774	0.05503	0.03774	0.03616	0.05503	
53_*An_tessellatus*_West_Pesisir_Lampung_Indonesia	0.05503	0.00472	0.00472	0.00472	0.00472	0.07862	0.00472	0.00629	0.07862	
50_*An_tessellatus*_Pulang_Pisau_Central_Kalimantan_Indonesia	0.01730	0.06447	0.06447	0.06447	0.06447	0.03774	0.06447	0.06289	0.03774	
MT257009.1_*An_tessellatus*_East_Kalimantan_1_Indonesia	0.02093	0.06924	0.06924	0.06924	0.06924	0.04831	0.06924	0.06763	0.04831	
MT257011.1_*An_tessellatus*_East_Kalimantan_2_Indonesia	0.01932	0.06763	0.06763	0.06763	0.06763	0.04509	0.06763	0.06602	0.04509	
MT257012.1_*An_tessellatus*_East_Kalimantan_3_Indonesia	0.06119	0.00000	0.00000	0.00000	0.00000	0.08535	0.00000	0.00161	0.08535	
MT257013.1_*An_tessellatus*_East_Kalimantan_4_Indonesia	0.02576	0.06441	0.06441	0.06441	0.06441	0.04992	0.06441	0.06280	0.04992	
MT753038.1_*An_tessellatus*_Sulawesi_Indonesia	0.05912	0.00000	0.00000	0.00000	0.00000	0.08108	0.00000	0.00169	0.08108	
MT256984.1_*An_tessellatus*_Sarawak_1_Malaysia	0.05410	0.00000	0.00000	0.00000	0.00000	0.07853	0.00000	0.00175	0.07853	
MT256985.1_*An_tessellatus*_Sarawak_2_Malaysia	0.05789	0.00351	0.00351	0.00351	0.00351	0.08070	0.00351	0.00526	0.08070	
MT256986.1_*An_tessellatus*_Sarawak_3_Malaysia	0.05272	0.00176	0.00176	0.00176	0.00176	0.07733	0.00176	0.00351	0.07733	
MT256990.1_*An_tessellatus*_Luzon_Philippines	0.06119	0.01127	0.01127	0.01127	0.01127	0.08696	0.01127	0.01288	0.08696	
MT256981.1_*An_tessellatus*_Mindanao_1_Philippines	0.02487	0.06217	0.06217	0.06217	0.06217	0.05329	0.06217	0.06394	0.05329	
MT257000.1_*An_tessellatus*_Mindanao_2_Philippines	0.03221	0.06280	0.06280	0.06280	0.06280	0.05636	0.06280	0.06441	0.05636	
MT257010.1_*An_tessellatus*_Mindanao_3_Philippines	0.02482	0.06206	0.06206	0.06206	0.06206	0.05319	0.06206	0.06383	0.05319	
MT257015.1_*An_tessellatus*_Mindanao_4_Philippines	0.02131	0.05861	0.05861	0.05861	0.05861	0.04973	0.05861	0.06039	0.04973	
*Anopheles tessellatus*	01_West_Sumatra_Indonesia	02_East_Nusa_Tenggara_Indonesia	05_Central_Sumba_East_Nusa_Tenggara_Indonesia	09_West_Lombok_West_Nusa_Tenggara_Indonesia	10_Bima_West_Nusa_Tenggara_Indonesia	12_Manado_North_Sulawesi_Indonesia	14_Pandawawi_East_Nusa_Tenggara_Indonesia	15_Matakapore_East_Nusa_Tenggara_Indonesia	17_Tojo_Una-Una_Central_Sulawesi_Indonesia	
KF564696.1_*An_tessellatus*_Singapore_1	0.02361	0.07725	0.07725	0.07725	0.07725	0.05794	0.07725	0.07511	0.05794	
KF564697.1_*An_tessellatus*_Singapore_2	0.01499	0.07923	0.07923	0.07923	0.07923	0.04711	0.07923	0.07709	0.04711	
KF564698.1_*An_tessellatus*_Singapore_3	0.01940	0.07759	0.07759	0.07759	0.07759	0.05388	0.07759	0.07543	0.05388	
KF564699.1_*An_tessellatus*_Singapore_4	0.01071	0.07923	0.07923	0.07923	0.07923	0.04497	0.07923	0.07709	0.04497	
MT257031.1_*An_tessellatus*_Laos	0.04509	0.04026	0.04026	0.04026	0.04026	0.06763	0.04026	0.04187	0.06763	
AB738146.1_*An_tessellatus*_Japan_1	0.04509	0.04348	0.04348	0.04348	0.04348	0.06763	0.04348	0.04187	0.06763	
AB738147.1_*An_tessellatus*_Japan_2	0.04509	0.04348	0.04348	0.04348	0.04348	0.06763	0.04348	0.04187	0.06763	
KX668149.1_*An_tessellatus*_Sri_Lanka_1	0.05324	0.06481	0.06481	0.06481	0.06481	0.07407	0.06481	0.06250	0.07407	
KX668150.1_*An_tessellatus*_Sri_Lanka_2	0.05324	0.06250	0.06250	0.06250	0.06250	0.07176	0.06250	0.06019	0.07176	
KX668151.1_*An_tessellatus*_Sri_Lanka_3	0.04662	0.05594	0.05594	0.05594	0.05594	0.06527	0.05594	0.05361	0.06527	
MT256975.1_*An_tessellatus*_Vietnam_1	0.04522	0.04000	0.04000	0.04000	0.04000	0.06783	0.04000	0.03826	0.06783	
MT256976.1_*An_tessellatus*_Vietnam_2	0.04720	0.04021	0.04021	0.04021	0.04021	0.06993	0.04021	0.03846	0.06993	
MT256978.1_*An_tessellatus*_Vietnam_4	0.04188	0.03665	0.03665	0.03665	0.03665	0.06283	0.03665	0.03490	0.06283	
OP477047.1_*An_tessellatus*_Thailand_1	0.04299	0.04459	0.04459	0.04459	0.04459	0.06529	0.04459	0.04299	0.06529	
ON533729.1_*An_tessellatus*_Thailand_2	0.04509	0.04348	0.04348	0.04348	0.04348	0.06763	0.04348	0.04187	0.06763	
ON533727.1_*An_tessellatus*_Thailand_3	0.04348	0.04509	0.04509	0.04509	0.04509	0.06602	0.04509	0.04348	0.06602	
ON533726.1_*An_tessellatus*_Thailand_4	0.04348	0.04509	0.04509	0.04509	0.04509	0.06602	0.04509	0.04348	0.06602	
ON533722.1_*An_tessellatus*_Thailand_5	0.04187	0.04348	0.04348	0.04348	0.04348	0.06602	0.04348	0.04187	0.06602	
*Anopheles tessellatus*	18_Banggai_1_Central_Sulawesi_Indonesia	20_Palu_1_Central_Sulawesi_Indonesia	36_Sigi_Central_Sulawesi_Indonesia	37_North_Morowali_Central_Sulawesi_Indonesia	38_Banggai_2_Central_Sulawesi_Indonesia	27_Kulon_Progo_1_Yogyakarta_Indonesia	87_Kulon_Progo_5_Yogyakarta_Indonesia	88_Kulon_Progo_6_Yogyakarta_Indonesia	42_Raja_Ampat_South_West_Papua_Indonesia	
18_*An_tessellatus*_Banggai_1_Central_Sulawesi_Indonesia										
20_*An_tessellatus*_Palu_1_Central_Sulawesi_Indonesia	0.00000									
36_*An_tessellatus*_Sigi_Central_Sulawesi_Indonesia	0.00000	0.00000								
37_*An_tessellatus*_North_Morowali_Central_Sulawesi_Indonesia	0.05818	0.05818	0.05818							
38_*An_tessellatus*_Banggai_2_Central_Sulawesi_Indonesia	0.00000	0.00000	0.00000	0.05818						
27_*An_tessellatus*_Kulon_Progo_1_Yogyakarta_Indonesia	0.00000	0.00000	0.00000	0.05818	0.00000					
87_*An_tessellatus*_Kulon_Progo_5_Yogyakarta_Indonesia	0.00000	0.00000	0.00000	0.05818	0.00000	0.00000				
88_*An_tessellatus*_Kulon_Progo_6_Yogyakarta_Indonesia	0.00000	0.00000	0.00000	0.05818	0.00000	0.00000	0.00000			
42_*An_tessellatus*_Raja_Ampat_South_West_Papua_Indonesia	0.00314	0.00314	0.00314	0.05660	0.00314	0.00314	0.00314	0.00314		
39_*An_tessellatus*_Manokwari_West_Papua_Indonesia	0.00314	0.00314	0.00314	0.05660	0.00314	0.00314	0.00314	0.00314	0.00000	
47_*An_tessellatus*_Garut_West_Java_Indonesia	0.03145	0.03145	0.03145	0.04088	0.03145	0.03145	0.03145	0.03145	0.02987	
43_*An_tessellatus*_Padang_West_Sumatra_Indonesia	0.05189	0.05189	0.05189	0.01730	0.05189	0.05189	0.05189	0.05189	0.05031	
85_*An_tessellatus*_Parigi_Moutong_Central_Sulawesi_Indonesia	0.03459	0.03459	0.03459	0.03774	0.03459	0.03459	0.03459	0.03459	0.03302	
46_*An_tessellatus*_Pangkajene_Island_South_Sulawesi_Indonesia	0.03774	0.03774	0.03774	0.03459	0.03774	0.03774	0.03774	0.03774	0.03774	
53_*An_tessellatus*_West_Pesisir_Lampung_Indonesia	0.00472	0.00472	0.00472	0.05346	0.00472	0.00472	0.00472	0.00472	0.00472	
50_*An_tessellatus*_Pulang_Pisau_Central_Kalimantan_Indonesia	0.06447	0.06447	0.06447	0.01258	0.06447	0.06447	0.06447	0.06447	0.06289	
MT257009.1_*An_tessellatus*_East_Kalimantan_1_Indonesia	0.06924	0.06924	0.06924	0.01288	0.06924	0.06924	0.06924	0.06924	0.06763	
MT257011.1_*An_tessellatus*_East_Kalimantan_2_Indonesia	0.06763	0.06763	0.06763	0.00805	0.06763	0.06763	0.06763	0.06763	0.06602	
MT257012.1_*An_tessellatus*_East_Kalimantan_3_Indonesia	0.00000	0.00000	0.00000	0.05958	0.00000	0.00000	0.00000	0.00000	0.00322	
MT257013.1_*An_tessellatus*_East_Kalimantan_4_Indonesia	0.06441	0.06441	0.06441	0.01449	0.06441	0.06441	0.06441	0.06441	0.06280	
MT753038.1_*An_tessellatus*_Sulawesi_Indonesia	0.00000	0.00000	0.00000	0.05743	0.00000	0.00000	0.00000	0.00000	0.00338	
MT256984.1_*An_tessellatus*_Sarawak_1_Malaysia	0.00000	0.00000	0.00000	0.05236	0.00000	0.00000	0.00000	0.00000	0.00349	
MT256985.1_*An_tessellatus*_Sarawak_2_Malaysia	0.00351	0.00351	0.00351	0.04912	0.00351	0.00351	0.00351	0.00351	0.00702	
MT256986.1_*An_tessellatus*_Sarawak_3_Malaysia	0.00176	0.00176	0.00176	0.05272	0.00176	0.00176	0.00176	0.00176	0.00527	
MT256990.1_*An_tessellatus*_Luzon_Philippines	0.01127	0.01127	0.01127	0.06119	0.01127	0.01127	0.01127	0.01127	0.01449	
MT256981.1_*An_tessellatus*_Mindanao_1_Philippines	0.06217	0.06217	0.06217	0.03375	0.06217	0.06217	0.06217	0.06217	0.06039	
MT257000.1_*An_tessellatus*_Mindanao_2_Philippines	0.06280	0.06280	0.06280	0.03704	0.06280	0.06280	0.06280	0.06280	0.06119	
MT257010.1_*An_tessellatus*_Mindanao_3_Philippines	0.06206	0.06206	0.06206	0.03369	0.06206	0.06206	0.06206	0.06206	0.06028	
MT257015.1_*An_tessellatus*_Mindanao_4_Philippines	0.05861	0.05861	0.05861	0.03020	0.05861	0.05861	0.05861	0.05861	0.05684	
KF564696.1_*An_tessellatus*_Singapore_1	0.07725	0.07725	0.07725	0.03004	0.07725	0.07725	0.07725	0.07725	0.07511	
KF564697.1_*An_tessellatus*_Singapore_2	0.07923	0.07923	0.07923	0.02998	0.07923	0.07923	0.07923	0.07923	0.07709	
KF564698.1_*An_tessellatus*_Singapore_3	0.07759	0.07759	0.07759	0.02155	0.07759	0.07759	0.07759	0.07759	0.07543	
KF564699.1_*An_tessellatus*_Singapore_4	0.07923	0.07923	0.07923	0.01713	0.07923	0.07923	0.07923	0.07923	0.07709	
*Anopheles tessellatus*	18_Banggai_1_Central_Sulawesi_Indonesia	20_Palu_1_Central_Sulawesi_Indonesia	36_Sigi_Central_Sulawesi_Indonesia	37_North_Morowali_Central_Sulawesi_Indonesia	38_Banggai_2_Central_Sulawesi_Indonesia	27_Kulon_Progo_1_Yogyakarta_Indonesia	87_Kulon_Progo_5_Yogyakarta_Indonesia	88_Kulon_Progo_6_Yogyakarta_Indonesia	42_Raja_Ampat_South_West_Papua_Indonesia	
MT257031.1_*An_tessellatus*_Laos	0.04026	0.04026	0.04026	0.04670	0.04026	0.04026	0.04026	0.04026	0.03865	
AB738146.1_*An_tessellatus*_Japan_1	0.04348	0.04348	0.04348	0.04670	0.04348	0.04348	0.04348	0.04348	0.04187	
AB738147.1_*An_tessellatus*_Japan_2	0.04348	0.04348	0.04348	0.04670	0.04348	0.04348	0.04348	0.04348	0.04187	
KX668149.1_*An_tessellatus*_Sri_Lanka_1	0.06481	0.06481	0.06481	0.05556	0.06481	0.06481	0.06481	0.06481	0.06250	
KX668150.1_*An_tessellatus*_Sri_Lanka_2	0.06250	0.06250	0.06250	0.05324	0.06250	0.06250	0.06250	0.06250	0.06019	
KX668151.1_*An_tessellatus*_Sri_Lanka_3	0.05594	0.05594	0.05594	0.04662	0.05594	0.05594	0.05594	0.05594	0.05361	
MT256975.1_*An_tessellatus*_Vietnam_1	0.04000	0.04000	0.04000	0.04348	0.04000	0.04000	0.04000	0.04000	0.03826	
MT256976.1_*An_tessellatus*_Vietnam_2	0.04021	0.04021	0.04021	0.04545	0.04021	0.04021	0.04021	0.04021	0.03846	
MT256978.1_*An_tessellatus*_Vietnam_4	0.03665	0.03665	0.03665	0.04014	0.03665	0.03665	0.03665	0.03665	0.03490	
OP477047.1_*An_tessellatus*_Thailand_1	0.04459	0.04459	0.04459	0.04459	0.04459	0.04459	0.04459	0.04459	0.04299	
ON533729.1_*An_tessellatus*_Thailand_2	0.04348	0.04348	0.04348	0.04670	0.04348	0.04348	0.04348	0.04348	0.04348	
ON533727.1_*An_tessellatus*_Thailand_3	0.04509	0.04509	0.04509	0.04509	0.04509	0.04509	0.04509	0.04509	0.04348	
ON533726.1_*An_tessellatus*_Thailand_4	0.04509	0.04509	0.04509	0.04509	0.04509	0.04509	0.04509	0.04509	0.04348	
ON533722.1_*An_tessellatus*_Thailand_5	0.04348	0.04348	0.04348	0.04348	0.04348	0.04348	0.04348	0.04348	0.04187	
*Anopheles tessellatus*	39_Manokwari_West_Papua_Indonesia	47_Garut_West_Java_Indonesia	43_Padang_West_Sumatra_Indonesia	85_Parigi_Moutong_Central_Sulawesi_Indonesia	46_Pangkajene_Island_South_Sulawesi_Indonesia	53_West_Pesisir_Lampung_Indonesia	50_Pulang_Pisau_Central_Kalimantan_Indonesia	MT257009.1_East_Kalimantan_1_Indonesia	MT257011.1_East_Kalimantan_2_Indonesia	
39_*An_tessellatus*_Manokwari_West_Papua_Indonesia										
47_*An_tessellatus*_Garut_West_Java_Indonesia	0.02987									
43_*An_tessellatus*_Padang_West_Sumatra_Indonesia	0.05031	0.02987								
85_*An_tessellatus*_Parigi_Moutong_Central_Sulawesi_Indonesia	0.03302	0.03145	0.02673							
46_*An_tessellatus*_Pangkajene_Island_South_Sulawesi_Indonesia	0.03774	0.03145	0.02358	0.01887						
53_*An_tessellatus*_West_Pesisir_Lampung_Indonesia	0.00472	0.02673	0.04717	0.02987	0.03302					
50_*An_tessellatus*_Pulang_Pisau_Central_Kalimantan_Indonesia	0.06289	0.03774	0.01730	0.04088	0.03774	0.05975				
MT257009.1_*An_tessellatus*_East_Kalimantan_1_Indonesia	0.06763	0.04509	0.02093	0.04509	0.04187	0.06441	0.01288			
MT257011.1_*An_tessellatus*_East_Kalimantan_2_Indonesia	0.06602	0.04348	0.01932	0.04348	0.04026	0.06280	0.01127	0.00483		
MT257012.1_*An_tessellatus*_East_Kalimantan_3_Indonesia	0.00322	0.03221	0.05314	0.03543	0.03865	0.00483	0.06602	0.06924	0.06991	
MT257013.1_*An_tessellatus*_East_Kalimantan_4_Indonesia	0.06280	0.04348	0.02576	0.04348	0.04026	0.05958	0.02093	0.01127	0.01216	
MT753038.1_*An_tessellatus*_Sulawesi_Indonesia	0.00338	0.03209	0.05068	0.03378	0.03716	0.00507	0.06419	0.06588	0.06677	
MT256984.1_*An_tessellatus*_Sarawak_1_Malaysia	0.00349	0.03141	0.04538	0.03141	0.03490	0.00524	0.05934	0.06108	0.06230	
MT256985.1_*An_tessellatus*_Sarawak_2_Malaysia	0.00702	0.03509	0.04561	0.03158	0.03509	0.00877	0.05965	0.06140	0.05931	
MT256986.1_*An_tessellatus*_Sarawak_3_Malaysia	0.00527	0.03163	0.04569	0.03163	0.03515	0.00703	0.05975	0.06151	0.06271	
MT256990.1_*An_tessellatus*_Luzon_Philippines	0.01449	0.04026	0.05475	0.04026	0.04026	0.01610	0.06441	0.06763	0.06839	
MT256981.1_*An_tessellatus*_Mindanao_1_Philippines	0.06039	0.04973	0.03020	0.04618	0.04618	0.05861	0.03375	0.03197	0.03333	
MT257000.1_*An_tessellatus*_Mindanao_2_Philippines	0.06119	0.05153	0.03865	0.04187	0.04509	0.05958	0.03704	0.04026	0.03799	
MT257010.1_*An_tessellatus*_Mindanao_3_Philippines	0.06028	0.04965	0.03014	0.04610	0.04610	0.05851	0.03723	0.03546	0.03661	
MT257015.1_*An_tessellatus*_Mindanao_4_Philippines	0.05684	0.04618	0.03020	0.04973	0.04618	0.05506	0.03375	0.03197	0.03333	
KF564696.1_*An_tessellatus*_Singapore_1	0.07511	0.05365	0.03648	0.05579	0.05150	0.07082	0.03219	0.03326	0.03104	
KF564697.1_*An_tessellatus*_Singapore_2	0.07709	0.04925	0.02355	0.04711	0.04283	0.07281	0.02355	0.01991	0.02212	
KF564698.1_*An_tessellatus*_Singapore_3	0.07543	0.05388	0.02802	0.04741	0.04310	0.07112	0.02371	0.02450	0.02227	
KF564699.1_*An_tessellatus*_Singapore_4	0.07709	0.05139	0.02141	0.04497	0.04069	0.07281	0.01499	0.01991	0.01327	
*Anopheles tessellatus*	39_Manokwari_West_Papua_Indonesia	47_Garut_West_Java_Indonesia	43_Padang_West_Sumatra_Indonesia	85_Parigi_Moutong_Central_Sulawesi_Indonesia	46_Pangkajene_Island_South_Sulawesi_Indonesia	53_West_Pesisir_Lampung_Indonesia	50_Pulang_Pisau_Central_Kalimantan_Indonesia	MT257009.1_East_Kalimantan_1_Indonesia	MT257011.1_East_Kalimantan_2_Indonesia	
MT257031.1_*An_tessellatus*_Laos	0.03865	0.04187	0.04348	0.04509	0.05153	0.03543	0.04992	0.04992	0.05319	
AB738146.1_*An_tessellatus*_Japan_1	0.04187	0.04187	0.04348	0.04509	0.05153	0.03865	0.04992	0.04992	0.05319	
AB738147.1_*An_tessellatus*_Japan_2	0.04187	0.04187	0.04348	0.04509	0.05153	0.03865	0.04992	0.04992	0.05319	
KX668149.1_*An_tessellatus*_Sri_Lanka_1	0.06250	0.05093	0.05324	0.05324	0.06481	0.05787	0.05324	0.05995	0.05516	
KX668150.1_*An_tessellatus*_Sri_Lanka_2	0.06019	0.04861	0.05093	0.05093	0.06250	0.05556	0.05093	0.05755	0.05276	
KX668151.1_*An_tessellatus*_Sri_Lanka_3	0.05361	0.04196	0.04429	0.04429	0.05594	0.04895	0.04429	0.05072	0.04589	
MT256975.1_*An_tessellatus*_Vietnam_1	0.03826	0.04000	0.04348	0.04348	0.05043	0.03478	0.05043	0.04870	0.05229	
MT256976.1_*An_tessellatus*_Vietnam_2	0.03846	0.04021	0.04021	0.04021	0.04720	0.03497	0.04545	0.04021	0.04762	
MT256978.1_*An_tessellatus*_Vietnam_4	0.03490	0.03665	0.04014	0.04014	0.04712	0.03141	0.04712	0.04538	0.04918	
OP477047.1_*An_tessellatus*_Thailand_1	0.04299	0.04299	0.04140	0.04299	0.04936	0.03981	0.04777	0.04831	0.05167	
ON533729.1_*An_tessellatus*_Thailand_2	0.04348	0.04509	0.04348	0.04509	0.04831	0.03865	0.04992	0.04992	0.05319	
ON533727.1_*An_tessellatus*_Thailand_3	0.04348	0.04348	0.04187	0.04348	0.04992	0.04026	0.04831	0.04831	0.05167	
ON533726.1_*An_tessellatus*_Thailand_4	0.04348	0.04348	0.04187	0.04348	0.04992	0.04026	0.04831	0.04831	0.05167	
ON533722.1_*An_tessellatus*_Thailand_5	0.04187	0.04187	0.04026	0.04187	0.04831	0.03865	0.04670	0.04670	0.05015	
*Anopheles tessellatus*	MT257012.1_East_Kalimantan_3_Indonesia	MT257013.1_East_Kalimantan_4_Indonesia	MT753038.1_Sulawesi_Indonesia	MT256984.1_Sarawak_1_Malaysia	MT256985.1_Sarawak_2_Malaysia	MT256986.1_Sarawak_3_Malaysia	MT256990.1_Luzon_Philippines	MT256981.1_Mindanao_1_Philippines	MT257000.1_Mindanao_2_Philippines	
MT257012.1_*An_tessellatus*_East_Kalimantan_3_Indonesia										
MT257013.1_*An_tessellatus*_East_Kalimantan_4_Indonesia	0.06687									
MT753038.1_*An_tessellatus*_Sulawesi_Indonesia	0.00000	0.06518								
MT256984.1_*An_tessellatus*_Sarawak_1_Malaysia	0.00000	0.06230	0.00000							
MT256985.1_*An_tessellatus*_Sarawak_2_Malaysia	0.00329	0.06260	0.00329	0.00329						
MT256986.1_*An_tessellatus*_Sarawak_3_Malaysia	0.00165	0.06271	0.00165	0.00165	0.00495					
MT256990.1_*An_tessellatus*_Luzon_Philippines	0.01064	0.06839	0.00954	0.00656	0.00988	0.00825				
MT256981.1_*An_tessellatus*_Mindanao_1_Philippines	0.06667	0.03667	0.06667	0.06667	0.06667	0.06667	0.06667			
MT257000.1_*An_tessellatus*_Mindanao_2_Philippines	0.06687	0.04103	0.06836	0.06721	0.06755	0.06601	0.07143	0.00667		
MT257010.1_*An_tessellatus*_Mindanao_3_Philippines	0.06656	0.03993	0.06656	0.06656	0.06656	0.06656	0.06988	0.00667	0.00998	
MT257015.1_*An_tessellatus*_Mindanao_4_Philippines	0.06333	0.03667	0.06333	0.06333	0.06667	0.06333	0.06667	0.01000	0.01333	
KF564696.1_*An_tessellatus*_Singapore_1	0.07539	0.03104	0.07820	0.07196	0.07250	0.07018	0.07761	0.03817	0.04213	
KF564697.1_*An_tessellatus*_Singapore_2	0.07743	0.02655	0.07565	0.06931	0.07481	0.06750	0.07522	0.03046	0.04204	
KF564698.1_*An_tessellatus*_Singapore_3	0.07572	0.02673	0.07857	0.07232	0.06784	0.07053	0.07795	0.02813	0.03341	
KF564699.1_*An_tessellatus*_Singapore_4	0.07743	0.02655	0.07565	0.06931	0.06484	0.06750	0.07522	0.02792	0.03319	
MT257031.1_*An_tessellatus*_Laos	0.04255	0.05319	0.04134	0.03770	0.03789	0.03960	0.05015	0.06000	0.06079	
AB738146.1_*An_tessellatus*_Japan_1	0.04559	0.05319	0.04452	0.04098	0.04119	0.04290	0.05319	0.06333	0.06383	
AB738147.1_*An_tessellatus*_Japan_2	0.04559	0.05319	0.04452	0.04098	0.04119	0.04290	0.05319	0.06333	0.06383	
KX668149.1_*An_tessellatus*_Sri_Lanka_1	0.06475	0.05995	0.06443	0.05962	0.06011	0.05753	0.06715	0.07242	0.06715	
KX668150.1_*An_tessellatus*_Sri_Lanka_2	0.06235	0.05755	0.06186	0.05691	0.05738	0.05479	0.06715	0.06964	0.06475	
KX668151.1_*An_tessellatus*_Sri_Lanka_3	0.05556	0.05072	0.05455	0.04918	0.04959	0.04696	0.06039	0.06180	0.05797	
MT256975.1_*An_tessellatus*_Vietnam_1	0.04248	0.05229	0.04248	0.04262	0.04283	0.04455	0.04902	0.06500	0.06536	
MT256976.1_*An_tessellatus*_Vietnam_2	0.04269	0.04762	0.04269	0.04269	0.04283	0.04455	0.04598	0.06000	0.06076	
MT256978.1_*An_tessellatus*_Vietnam_4	0.03934	0.04918	0.03934	0.03934	0.03954	0.04125	0.04590	0.06167	0.06230	
OP477047.1_*An_tessellatus*_Thailand_1	0.04711	0.05167	0.04452	0.04098	0.04119	0.04290	0.05167	0.06333	0.06535	
ON533729.1_*An_tessellatus*_Thailand_2	0.04559	0.05319	0.04293	0.03934	0.03954	0.04125	0.05015	0.06500	0.06687	
ON533727.1_*An_tessellatus*_Thailand_3	0.04711	0.05167	0.04452	0.04098	0.04119	0.04290	0.05167	0.06333	0.06535	
ON533726.1_*An_tessellatus*_Thailand_4	0.04711	0.05167	0.04452	0.04098	0.04119	0.04290	0.05167	0.06333	0.06535	
ON533722.1_*An_tessellatus*_Thailand_5	0.04559	0.05015	0.04293	0.03934	0.03954	0.04125	0.05015	0.06167	0.06383	
*Anopheles tessellatus*	MT257010.1_Mindanao_3_Philippines	MT257015.1_Mindanao_4_Philippines	KF564696.1_Singapore_1	KF564697.1_Singapore_2	KF564698.1_Singapore_3	KF564699.1_Singapore_4	MT257031.1_Laos	AB738146.1_Japan_1	AB738147.1_Japan_2	KX668149.1_Sri_Lanka_1
MT257010.1_*An_tessellatus*_Mindanao_3_Philippines										
MT257015.1_*An_tessellatus*_Mindanao_4_Philippines	0.00667									
KF564696.1_*An_tessellatus*_Singapore_1	0.04569	0.04071								
KF564697.1_*An_tessellatus*_Singapore_2	0.03797	0.03299	0.02146							
KF564698.1_*An_tessellatus*_Singapore_3	0.03571	0.03581	0.01296	0.01293						
KF564699.1_*An_tessellatus*_Singapore_4	0.03038	0.03046	0.01717	0.01499	0.01078					
MT257031.1_*An_tessellatus*_Laos	0.05657	0.05333	0.05543	0.05752	0.05568	0.05088				
AB738146.1_*An_tessellatus*_Japan_1	0.05990	0.05667	0.05543	0.05752	0.05568	0.05088	0.00304			
AB738147.1_*An_tessellatus*_Japan_2	0.05990	0.05667	0.05543	0.05752	0.05568	0.05088	0.00304	0.00000		
KX668149.1_*An_tessellatus*_Sri_Lanka_1	0.07222	0.06685	0.06032	0.06250	0.05787	0.05556	0.03357	0.03357	0.03357	
KX668150.1_*An_tessellatus*_Sri_Lanka_2	0.06944	0.06407	0.06032	0.06250	0.05787	0.05556	0.03118	0.03118	0.03118	0.00231
KX668151.1_*An_tessellatus*_Sri_Lanka_3	0.06162	0.05618	0.05374	0.05594	0.05128	0.04895	0.02415	0.02415	0.02415	0.00699
MT256975.1_*An_tessellatus*_Vietnam_1	0.06156	0.05833	0.05679	0.05911	0.05707	0.05172	0.00817	0.00490	0.00490	0.03235
MT256976.1_*An_tessellatus*_Vietnam_2	0.05990	0.06000	0.05970	0.04963	0.05250	0.05211	0.00985	0.00985	0.00985	0.04348
MT256978.1_*An_tessellatus*_Vietnam_4	0.05824	0.05500	0.05211	0.05446	0.05237	0.04703	0.00164	0.00164	0.00164	0.03252
OP477047.1_*An_tessellatus*_Thailand_1	0.05990	0.05667	0.05677	0.05447	0.05702	0.04793	0.00456	0.00152	0.00152	0.03538
ON533729.1_*An_tessellatus*_Thailand_2	0.06156	0.05833	0.05987	0.05752	0.06013	0.05088	0.00608	0.00304	0.00304	0.03837
ON533727.1_*An_tessellatus*_Thailand_3	0.05990	0.05667	0.05765	0.05531	0.05791	0.04867	0.00456	0.00152	0.00152	0.03597
ON533726.1_*An_tessellatus*_Thailand_4	0.05990	0.05667	0.05765	0.05531	0.05791	0.04867	0.00456	0.00152	0.00152	0.03597
ON533722.1_*An_tessellatus*_Thailand_5	0.05824	0.05500	0.05543	0.05310	0.05568	0.04646	0.00608	0.00304	0.00304	0.03357
*Anopheles tessellatus*	KX668150.1_Sri_Lanka_2	KX668151.1_Sri_Lanka_3	MT256975.1_Vietnam_1	MT256976.1_Vietnam_2	MT256978.1_Vietnam_4	OP477047.1_Thailand_1	ON533729.1_Thailand_2	ON533727.1_Thailand_3	ON533726.1_Thailand_4	ON533722.1_Thailand_5
KX668150.1_*An_tessellatus*_Sri_Lanka_2										
KX668151.1_*An_tessellatus*_Sri_Lanka_3	0.00466									
MT256975.1_*An_tessellatus*_Vietnam_1	0.02965	0.02717								
MT256976.1_*An_tessellatus*_Vietnam_2	0.04076	0.03288	0.01478							
MT256978.1_*An_tessellatus*_Vietnam_4	0.02981	0.02186	0.00656	0.00821						
OP477047.1_*An_tessellatus*_Thailand_1	0.03302	0.02613	0.00490	0.00985	0.00164					
ON533729.1_*An_tessellatus*_Thailand_2	0.03597	0.02899	0.00654	0.01149	0.00328	0.00152				
ON533727.1_*An_tessellatus*_Thailand_3	0.03357	0.02657	0.00490	0.00985	0.00164	0.00000	0.00152			
ON533726.1_*An_tessellatus*_Thailand_4	0.03357	0.02657	0.00490	0.00985	0.00164	0.00000	0.00152	0.00000		
ON533722.1_*An_tessellatus*_Thailand_5	0.03118	0.02415	0.00327	0.01149	0.00328	0.00152	0.00304	0.00152	0.00152	

**Table 5:** Mean genetic distances within (diagonal) and between (off-diagonal) COI lineages estimated using the Kimura 2-parameter model.

Lineage	1	2	3	4	5	6	7	8	9	10	11	12
**1**	**0.0022 ± 0.0006**	0.0057	0.0326	0.0351	0.0385	0.0528	0.0658	0.0878	0.0775	0.0669	0.0625	0.0439
**2**		**n/c**	0.0272	0.0305	0.0339	0.0488	0.0604	0.0833	0.0724	0.0627	0.0562	0.0384
**3**			**n/c**	0.0322	0.0322	0.0305	0.0512	0.0539	0.0517	0.0435	0.0487	0.0431
**4**				**n/c**	0.0191	0.0273	0.0476	0.059	0.0483	0.0435	0.0513	0.0448
**5**					**n/c**	0.024	0.0474	0.0573	0.0439	0.04	0.0638	0.0514
**6**						**n/c**	0.0332	0.0507	0.0253	0.0205	0.0512	0.0435
**7**							**0.0090 ± 0.0029**	0.0555	0.0344	0.0368	0.0687	0.0638
**8**								**0.0000 ± 0.0000**	0.0512	0.0464	0.074	0.0702
**9**									**0.0166 ± 0.0044**	0.0241	0.0579	0.0546
**10**										**0.0123 ± 0.0031**	0.055	0.0507
**11**											**0.0047 ± 0.0026**	0.0322
**12**												**0.0043 ± 0.0013**

**Note:** Values on the diagonal represent the mean genetic distance within lineages (mean ± SE), whereas values above the diagonal represent the mean genetic distance between lineages. n/c = not calculated because only a single sequence was available in the lineage.

**Fig 5:**
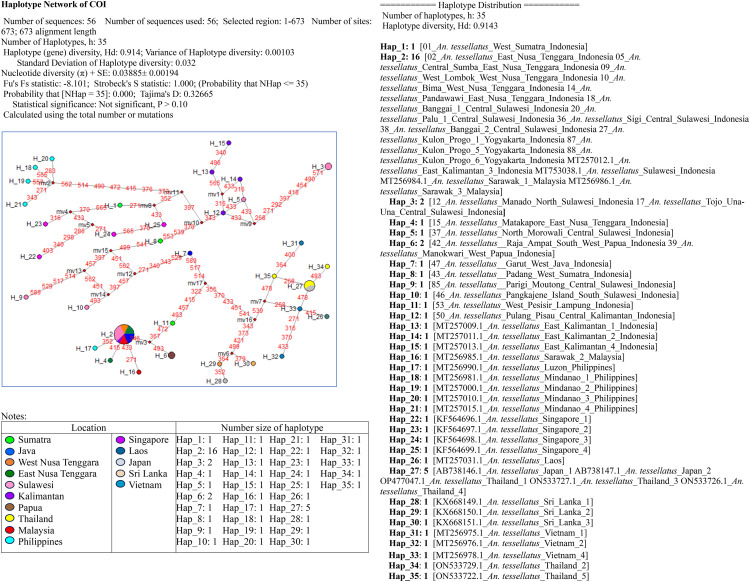
Haplotype network of *Anopheles tessellatus* based on COI sequences. The Fig depicts the frequency of each COI haplotype across the study sites, with lines indicating the number of mutational steps separating haplotypes and their geographic distribution.

Subsequently, COI haplotype analysis of *An. tessellatus* demonstrates exceptionally high genetic diversity, with 35 haplotypes identified from approximately 54 sequences across 56 variable sites, resulting a haplotype diversity (Hd) of 0.914 (Fig 5). This high level of haplotype diversity underscores the sensitivity of the mitochondrial COI marker to recent demographic processes in mosquito populations and exceeds the diversity detected using ITS2, thereby reaffirming the utility of COI for detecting cryptic genetic structure within *An. tessellatus* across Southeast Asia [2]. Hap2 was the most dominant haplotype (n=16), encompassing Indonesian samples from East Nusa Tenggara, West Nusa Tenggara, Central Sulawesi, Yogyakarta, as well as references from East Kalimantan (Indonesia) and Sarawak (Malaysia). This haplotype is defined by mutations at positions 2-5, 7, 10-12, 14-17, 28, 30-31, and 33, indicating a widespread, possibly ancestral clade with ongoing gene flow [6]. Hap27 (n=5) clusters Japanese and Thai references (positions 44-45, 52, 54-55), while singleton haplotypes (e.g., Hap1 from West Sumatra; Hap3 n=2 from North/Central Sulawesi at sites 6,9) predominate, alongside unique regional variants from Papua, West Java, Lampung, Kalimantan, Philippines, Singapore, Sri Lanka, Vietnam, and Laos, underscoring pan-Asian phylogeographic structuring. In median‑joining or statistical parsimony networks, Hap2 is typically positioned as a central node, connected by short mutational branches to Indonesian singletons (Hap 4-6, 9-12), reflecting low divergence and substantial connectivity likely facilitated by island dispersal or anthropogenic movement. In contrast, longer branches leading to Hap27 (Thailand/Japan) and to more distantly related Asian haplotypes (e.g., Hap 28-35), implying historical vicariance or isolation by distance. His star-like topology with a dominant hub suggests recent demographic expansion in Indonesian populations, contrasting lower ITS2 diversity and aligning with neutral evolution under Tajima’s D neutrality tests for vector surveillance (Fig 5) [2].

### 3.3. The Cytochrome Oxidase II gene (COII) diversity, phylogeny, and polymorphism of *Anopheles tessellatus*

**Fig 6:**
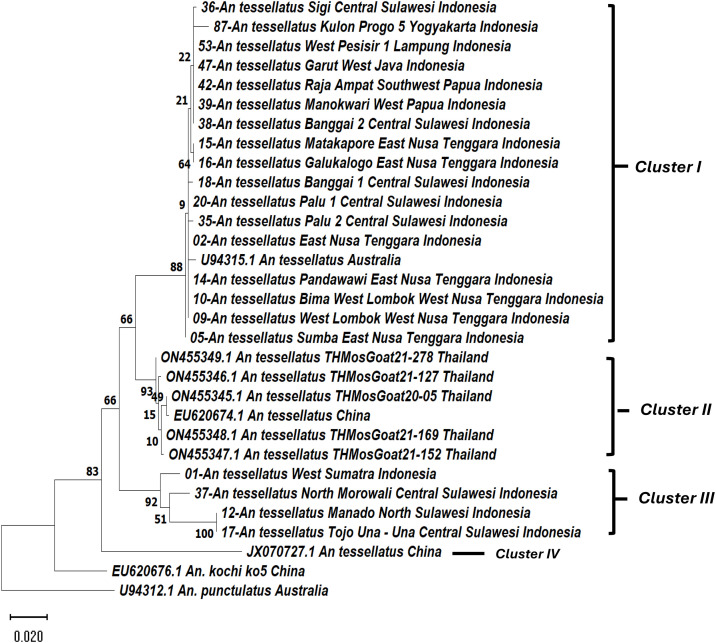
Phylogenetic analysis of COII sequences of *Anopheles tessellatus*, rooted with *Anopheles punctulatus* as the outgroup. The phylogenetic tree was constructed using the General Time Reversible (GTR+I) model in MEGA X, with bootstrap support estimated from 1,000 replicates to assess tree reliability.

Analysis of COII sequences from 21 *An. tessellatus* specimens collected in this study (18 locations) across Indonesia, along with eight reference sequences retrieved from GenBank (one from Australia, two from China, and five from Thailand), revealed four distinct population clusters (Tables 6, 7). Cluster I comprised specimens from West Pesisir (Lampung) (PX 115250), Sigi, Banggai, and Palu (Central Sulawesi) (PX115252, PX115247, PX115254, PX115248, PX115251), Garut (West Java) (PX115257), Kulon Progo (Yogyakarta) (PX115262), West Lombok and Bima (West Nusa Tenggara) (PX115240-PX115241), Pandawawi, Matakapore, Galukalogo, and Central Sumba (East Nusa Tenggara) (PX115243-PX115245, PX115239), Manokwari (PX115255), Raja Ampat (Southwest Papua) (PX115256), and one sample of GenBank reference from Australia (U94315). Cluster II consisted of GenBank reference samples from Thailand (ON455345-ON455349) and China (EU620674). Cluster III contained specimens from West Sumatra (PX115250), North Morowali (Central Sulawesi) (PX115253), Tojo Una-Una (Central Sulawesi) (PX115246), and Manado (North Sulawesi) (PX115242), Cluster IV included a single GenBank reference sequence from China (JX070727) (Fig 6; Tables 1, 7).

**Table 6:** Genetic distance of *Anopheles tessellatus* based on the COII gene in Indonesia using p-distance.

*Anopheles tessellatus*	01_West_Sumatra_Indonesia	02_East_Nusa_Tenggara_Indonesia	05_Sumba_East_Nusa_Tenggara_Indonesia	09_West_Lombok_West_Nusa_Tenggara_Indonesia	10_Bima_West_Lombok_West_Nusa_Tenggara_Indonesia	12_Manado_North_Sulawesi_Indonesia	14_Pandawawi_East_Nusa_Tenggara_Indonesia	15_Matakapore_East_Nusa_Tenggara_Indonesia	16_Galukalogo_East_Nusa_Tenggara_Indonesia		
01_*An_tessellatus*_West_Sumatra_Indonesia											
02_*An_tessellatus*_East_Nusa_Tenggara_Indonesia	0.05087										
05_*An_tessellatus*_Sumba_East_Nusa_Tenggara_Indonesia	0.04928	0.00137									
09_*An_tessellatus*_West_Lombok_West_Nusa_Tenggara_Indonesia	0.05087	0.00000	0.00140								
10_*An_tessellatus*_Bima_West_Lombok_West_Nusa_Tenggara_Indonesia	0.05087	0.00000	0.00149	0.00000							
12_*An_tessellatus*_Manado_North_Sulawesi_Indonesia	0.03339	0.06421	0.06284	0.06573	0.06696						
14_*An_tessellatus*_Pandawawi_East_Nusa_Tenggara_Indonesia	0.05087	0.00000	0.00137	0.00000	0.00000	0.06421					
15 *An_tessellatus*_Matakapore_East_Nusa_Tenggara_Indonesia	0.05246	0.00273	0.00410	0.00280	0.00298	0.06284	0.00273				
16_*An_tessellatus*_Galukalogo_East_Nusa_Tenggara_Indonesia	0.05246	0.00273	0.00410	0.00280	0.00298	0.06284	0.00273	0.00000			
17_*An_tessellatus*_Tojo_Una_Una_Central_Sulawesi_Indonesia	0.03339	0.06421	0.06284	0.06573	0.06696	0.00000	0.06421	0.06284	0.06284		
18_*An_tessellatus*_Banggai_1_Central_Sulawesi_Indonesia	0.05405	0.00273	0.00410	0.00280	0.00298	0.06421	0.00273	0.00546	0.00546		
20_*An_tessellatus*_Palu_1_Central_Sulawesi_Indonesia	0.05087	0.00000	0.00149	0.00000	0.00000	0.06696	0.00000	0.00298	0.00298		
35_*An_tessellatus*_Palu_2_Central_Sulawesi_Indonesia	0.05087	0.00297	0.00446	0.00297	0.00298	0.06686	0.00297	0.00594	0.00594		
36_*An_tessellatus*_Sigi_Central_Sulawesi_Indonesia	0.05455	0.00545	0.00727	0.00545	0.00545	0.06364	0.00545	0.00545	0.00545		
37_*An_tessellatus*_North_Morowali_Central_Sulawesi_Indonesia	0.02373	0.05763	0.05593	0.05763	0.05763	0.03220	0.05763	0.05593	0.05593		
38_*An_tessellatus*_Banggai_2_Central_Sulawesi_Indonesia	0.05405	0.00273	0.00410	0.00280	0.00298	0.06148	0.00273	0.00273	0.00273		
39_*An_tessellatus*_Manokwari_West_Papua_Indonesia	0.05405	0.00273	0.00410	0.00280	0.00298	0.06148	0.00273	0.00273	0.00273		
42_*An_tessellatus*_Raja_Ampat_Southwest_Papua_Indonesia	0.05405	0.00273	0.00410	0.00280	0.00298	0.06148	0.00273	0.00273	0.00273		
47_*An_tessellatus*_Garut_West_Java_Indonesia	0.05405	0.00273	0.00410	0.00280	0.00298	0.06148	0.00273	0.00273	0.00273		
53_*An_tessellatus*_West_Pesisir_1_Lampung_Indonesia	0.05405	0.00273	0.00410	0.00280	0.00298	0.06148	0.00273	0.00273	0.00273		
87_*An_tessellatus*_Kulon_Progo_5_Yogyakarta_Indonesia	0.05751	0.01097	0.00960	0.01124	0.01196	0.06447	0.01097	0.01097	0.01097		
U94315.1_*An_tessellatus*_Australia	0.05074	0.00439	0.00585	0.00439	0.00459	0.06871	0.00439	0.00731	0.00731		
ON455345.1_*An_tessellatus*_THMosGoat20-05_Thailand	0.04235	0.03704	0.03561	0.03714	0.03805	0.06125	0.03704	0.03704	0.03704		
JX070727.1_*An_tessellatus*_China	0.08940	0.08811	0.08664	0.08811	0.09119	0.09838	0.08811	0.08811	0.08811		
EU620674.1_*An_tessellatus*_China	0.04092	0.03942	0.03796	0.03942	0.03976	0.06277	0.03942	0.03942	0.03942		
ON455349.1_*An_tessellatus*_THMosGoat21-278_Thailand	0.04072	0.03704	0.03561	0.03714	0.03805	0.05983	0.03704	0.03704	0.03704		
ON455348.1_*An_tessellatus*_THMosGoat21-169_Thailand	0.04397	0.03704	0.03561	0.03714	0.03805	0.06125	0.03704	0.03704	0.03704		
ON455347.1_*An_tessellatus*_THMosGoat21-152_Thailand	0.04560	0.03561	0.03419	0.03571	0.03653	0.05983	0.03561	0.03561	0.03561		
ON455346.1_*An_tessellatus*_THMosGoat21-127_Thailand	0.04397	0.03704	0.03561	0.03714	0.03805	0.05983	0.03704	0.03704	0.03704		
*Anopheles tessellatus*	17_Tojo_Una_Una_Central_Sulawesi_Indonesia	18_Banggai_1_Central_Sulawesi_Indonesia	20_Palu_1_Central_Sulawesi_Indonesia	35_Palu_2_Central_Sulawesi_Indonesia	36_Sigi_Central_Sulawesi_Indonesia	37_North_Morowali_Central_Sulawesi_Indonesia	38_Banggai_2_Central_Sulawesi_Indonesia	39_Manokwari_West_Papua_Indonesia	42_Raja_Ampat_Southwest_Papua_Indonesia		
01_*An_tessellatus*_West_Sumatra_Indonesia											
02_*An_tessellatus*_East_Nusa_Tenggara_Indonesia											
05_*An_tessellatus*_Sumba_East_Nusa_Tenggara_Indonesia											
09_*An_tessellatus*_West_Lombok_West_Nusa_Tenggara_Indonesia											
10_*An_tessellatus*_Bima_West_Lombok_West_Nusa_Tenggara_Indonesia											
12_*An_tessellatus*_Manado_North_Sulawesi_Indonesia											
14_*An_tessellatus*_Pandawawi_East_Nusa_Tenggara_Indonesia											
15 *An_tessellatus*_Matakapore_East_Nusa_Tenggara_Indonesia											
16_*An_tessellatus*_Galukalogo_East_Nusa_Tenggara_Indonesia											
17_*An_tessellatus*_Tojo_Una_Una_Central_Sulawesi_Indonesia											
18_*An_tessellatus*_Banggai_1_Central_Sulawesi_Indonesia	0.06421										
20_*An_tessellatus*_Palu_1_Central_Sulawesi_Indonesia	0.06696	0.00298									
35_*An_tessellatus*_Palu_2_Central_Sulawesi_Indonesia	0.06686	0.00594	0.00298								
36_*An_tessellatus*_Sigi_Central_Sulawesi_Indonesia	0.06364	0.00909	0.00545	0.00545							
37_*An_tessellatus*_North_Morowali_Central_Sulawesi_Indonesia	0.03220	0.06102	0.05763	0.05763	0.05636						
38_*An_tessellatus*_Banggai_2_Central_Sulawesi_Indonesia	0.06148	0.00546	0.00298	0.00594	0.00182	0.05424					
39_*An_tessellatus*_Manokwari_West_Papua_Indonesia	0.06148	0.00546	0.00298	0.00594	0.00182	0.05424	0.00000				
42_*An_tessellatus*_Raja_Ampat_Southwest_Papua_Indonesia	0.06148	0.00546	0.00298	0.00594	0.00182	0.05424	0.00000	0.00000			
47_*An_tessellatus*_Garut_West_Java_Indonesia	0.06148	0.00546	0.00298	0.00594	0.00182	0.05424	0.00000	0.00000	0.00000		
53_*An_tessellatus*_West_Pesisir_1_Lampung_Indonesia	0.06148	0.00546	0.00298	0.00594	0.00182	0.05424	0.00000	0.00000	0.00000		
87_*An_tessellatus*_Kulon_Progo_5_Yogyakarta_Indonesia	0.06447	0.01097	0.01196	0.01493	0.01280	0.04770	0.00823	0.00823	0.00823		
U94315.1_*An_tessellatus*_Australia	0.06871	0.00731	0.00459	0.00763	0.01128	0.06119	0.00731	0.00731	0.00731		
ON455345.1_*An_tessellatus*_THMosGoat20-05_Thailand	0.06125	0.03989	0.03805	0.04103	0.03364	0.04696	0.03419	0.03419	0.03419		
JX070727.1_*An_tessellatus*_China	0.09838	0.09104	0.09119	0.09414	0.09905	0.08673	0.08811	0.08811	0.08811		
EU620674.1_*An_tessellatus*_China	0.06277	0.04234	0.03976	0.04275	0.03571	0.04545	0.03650	0.03650	0.03650		
ON455349.1_*An_tessellatus*_THMosGoat21-278_Thailand	0.05983	0.03989	0.03805	0.04103	0.03551	0.04870	0.03419	0.03419	0.03419		
ON455348.1_*An_tessellatus*_THMosGoat21-169_Thailand	0.06125	0.03989	0.03805	0.04103	0.03551	0.04870	0.03419	0.03419	0.03419		
ON455347.1_*An_tessellatus*_THMosGoat21-152_Thailand	0.05983	0.03846	0.03653	0.03951	0.03551	0.04870	0.03276	0.03276	0.03276		
ON455346.1_*An_tessellatus*_THMosGoat21-127_Thailand	0.05983	0.03989	0.03805	0.04103	0.03551	0.04522	0.03419	0.03419	0.03419		
*Anopheles tessellatus*	47_Garut_West_Java_Indonesia	53_West_Pesisir_1_Lampung_Indonesia	87_Kulon_Progo_5_Yogyakarta_Indonesia	U94315.1_Australia	ON455345.1_THMosGoat20-05_Thailand	JX070727.1_China	EU620674.1_China	ON455349.1_THMosGoat21-278_Thailand	ON455348.1_THMosGoat21-169_Thailand	ON455347.1_THMosGoat21-152_Thailand	ON455346.1_THMosGoat21-127_Thailand
47_*An_tessellatus*_Garut_West_Java_Indonesia											
53_*An_tessellatus*_West_Pesisir_1_Lampung_Indonesia	0.00000										
87_*An_tessellatus*_Kulon_Progo_5_Yogyakarta_Indonesia	0.00823	0.00823									
U94315.1_*An_tessellatus*_Australia	0.00731	0.00731	0.01608								
ON455345.1_*An_tessellatus*_THMosGoat20-05_Thailand	0.03419	0.03419	0.02849	0.03655							
JX070727.1_*An_tessellatus*_China	0.08811	0.08811	0.08223	0.08863	0.07342						
EU620674.1_*An_tessellatus*_China	0.03650	0.03650	0.03066	0.03801	0.00146	0.07227					
ON455349.1_*An_tessellatus*_THMosGoat21-278_Thailand	0.03419	0.03419	0.03134	0.03655	0.00570	0.07342	0.00438				
ON455348.1_*An_tessellatus*_THMosGoat21-169_Thailand	0.03419	0.03419	0.02849	0.03655	0.00285	0.07342	0.00438	0.00285			
ON455347.1_*An_tessellatus*_THMosGoat21-152_Thailand	0.03276	0.03276	0.02707	0.03509	0.00427	0.07195	0.00584	0.00427	0.00142		
											

**Table 7:** Mean genetic distances within (diagonal) and between (off-diagonal) COII clusters of *An. tessellatus* estimated using the Kimura 2-parameter model.

Cluster	Cluster I	Cluster II	Cluster III	Cluster IV
Cluster I	**0,0042 ± 0,0013**	0,0373	0,0620	0,0952
Cluster II		**0,0038 ± 0,0016**	0,0551	0,0769
Cluster III			**0,0265 ± 0,0054**	0,0999
Cluster IV				**n/c**

Notes: Diagonal values represent mean within-cluster genetic distances, whereas values below the diagonal represent mean genetic distances between clusters estimated using the Kimura 2-parameter (K2P) model. n/c = not calculated.

Genetic distances among members of Cluster I ranged from 0.02% to 0.13%, whereas intra-cluster COII sequence divergence within Cluster II ranged from 0.01% to 0.06%. In Cluster III, genetic distances were higher, ranging from 0.24% to 0.33%. Overall COII divergence among all samples ranged from 0.03% to 0.36%. The COII phylogenetic analysis revealed a simpler clustering pattern that was nonetheless congruent with the COI-based phylogeny, indicating that *An. tessellatus* populations distributed across Indonesia remain within the same maternal lineage despite long-term geographic isolation among islands and regions. The greater apparent complexity of the COI analysis likely reflects the larger number and broader taxonomic and geographic representation of *An. tessellatus* references available in GenBank for COI compared with COII (Fig 6; Table 7; S3 Fig).

**Fig 7:**
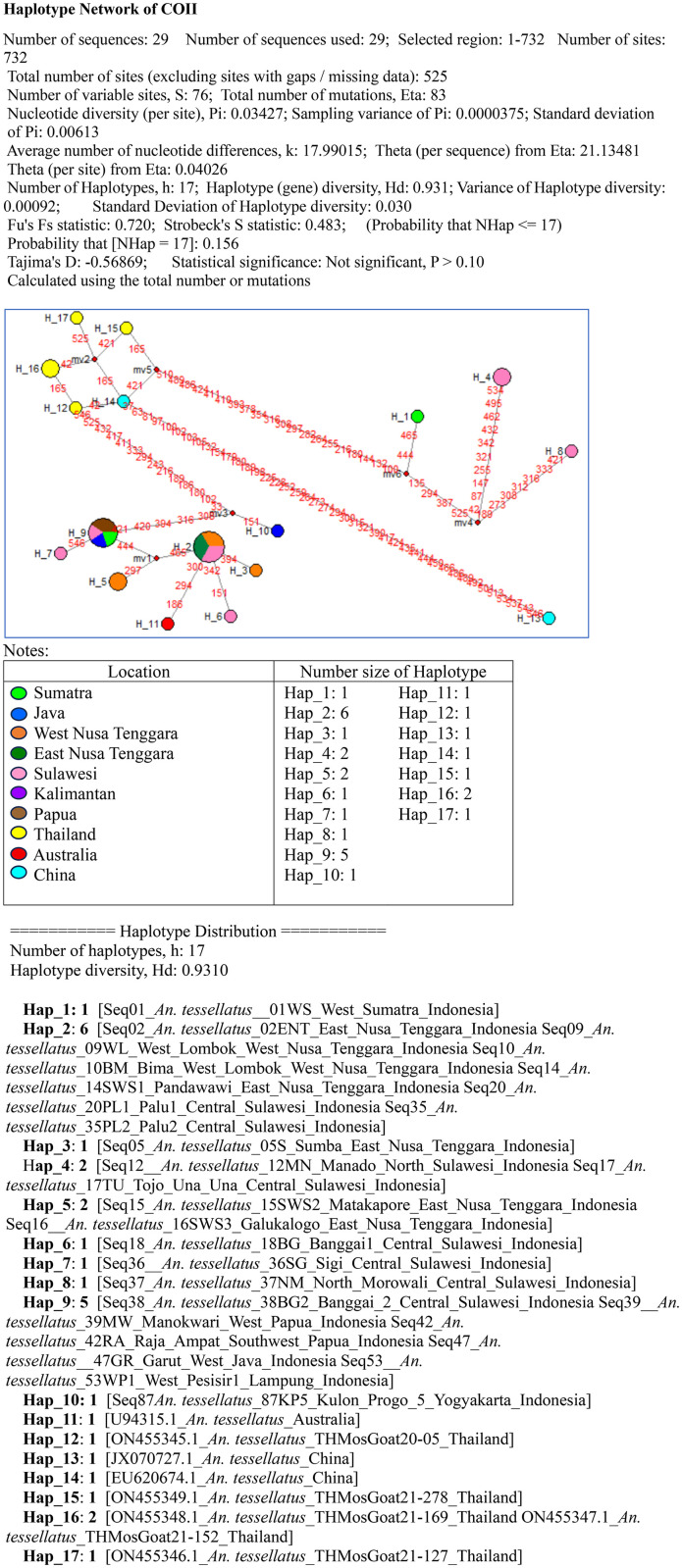
Haplotype network of *Anopheles tessellatus* based on COII sequences. The Fig shows the frequency of each COII haplotype across the study sites, with connecting lines representing the number of mutational steps separating haplotypes and their geographic distribution.

Furthermore, COII haplotype analysis of *An. tessellatus* based on 29 Indonesian sequences (525 bp alignment, 76 variable sites) reveals high genetic diversity, with 17 haplotypes and haplotype diversity (Hd) of 0.931 (Fig 7), underscoring this mitochondrial gene’s utility for resolving population structure in Southeast Asian mosquito vectors. Compared to COI (Hd=0.914, 35 haplotypes) and ITS2 (Hd=0.716, 5 haplotypes), COII shows intermediate resolution, reflecting concerted mtDNA evolution with elevated polymorphism suitable for phylogeographic inference [2]. Hap2 (n=6) predominates among Indonesian samples from East Nusa Tenggara, West Nusa Tenggara, and Central Sulawesi (positions 2, 4-5, 7, 12-13), indicating a core clade with regional connectivity. Hap9 (n=5) links Central Sulawesi, West Papua, West Java, and Lampung (positions 16-20), while Hap4 (n=2, North/Central Sulawesi at 6,10), and Hap5 (n=2, East Nusa Tenggara at 8-9) show localized clustering; singletons like Hap1 (West Sumatra), and Hap10 (Yogyakarta) highlight site-specific variants. Reference sequences from distinct groups: Hap11 (Australia), Hap12-17 (Thailand/China), suggesting geographic barriers or historical divergence from Indonesian populations [32]. Haplotype networks in this study would feature Hap2 as a central hub with short branches to Indonesian singletons (Hap 3,5-10), evidencing recent gene flow and star-like expansion across archipelago islands, while longer branches connect to Thailand/Chinese haplotypes (Hap12-17), implying isolation by distance or Pleistocene vicariance (Fig 7). This topology, with low reticulation, aligns with neutrality, indices signalling demographic growth, contrasting nuclear ITS2’s shallower structure, and supporting mtDNA’s maternal lineage tracking for vector dispersal models. We did not calculate site-by-site FSTF_{ST}FST/KSTK_{ST}KST for locations with singleton sampling because such estimates are unreliable. We grouped samples into broader geographic regions where biologically defensible and sample sizes were less sparse.

## 4. Discussion

### 4.1. Genetic structuring and lineage differentiation in *Anopheles tessellatus* across Indonesia

This study provides a comprehensive molecular assessment of *An. tessellatus* populations across Indonesia using ITS2 nuclear and COI, COII mitochondrial markers, revealing clear evidence of genetic structuring and geographically associated lineages within what has traditionally been regarded as a single species. Although all analysed populations form a monophyletic group, phylogenetic reconstruction and genetic distance analyses consistently identify three principal lineages, broadly corresponding to Sumatra, Sulawesi, and Java–Nusa Tenggara. These findings support the hypothesis that *An. tessellatus* in Indonesia represents a genetically structured species complex undergoing intraspecific diversification, and possibly speciation due to geographic distances between these lineages. The concordant clustering patterns observed across nuclear and mitochondrial loci indicate that the detected structure reflects genuine evolutionary differentiation rather than stochastic variation or marker-specific bias. Similar multilocus congruence has been widely interpreted as evidence of lineage divergence in other *Anopheles* complexes, including *An. dirus*, *An. sundaicus*, and *An. maculipennis* [10,30,33].

According to some sources, Indonesian populations of *An. tessellatus* exhibit significant morphological variations, with at least 11 morphological variants and three officially recognized subspecies. However, the present study does not allow to directly determine whether the three molecular lineages identified here correspond to any of these named forms, as the sequenced specimens were not re-evaluated according to a standardized morphological framework based on reference specimens. Therefore, the observed lineages should currently be interpreted as genetically differentiated populations within the *An. tessellatus* complex rather than as a confirmed subspecies. Future integrative taxonomic studies that combine morphology, molecular markers, and ecological data are needed to test whether these lineages represent the same entities as the recognized subspecies or morphological variants. Among the currently recognized subspecies, *An. tessellatus orientalis* has historically been reported from Sulawesi and other eastern Indonesian islands, which makes it a plausible candidate for comparison with the Sulawesi-associated lineage in our dataset.

### 4.2. Comparative performance of ITS2, COI, and COII markers

The three genetic markers employed in this study exhibited complementary resolution. The nuclear ITS2 region showed moderate haplotype diversity and relatively low nucleotide divergence, consistent with the effects of concerted evolution acting on ribosomal DNA arrays [16]. This pattern indicates that the nuclear ITS2 marker is more conserved and therefore less sensitive to recent population differentiation. Despite this evolutionary constraint, ITS2 successfully discriminated multiple population clusters, demonstrating its utility for identifying higher-level genetic structure within *An. tessellatus* (S1 Table).

In contrast, mitochondrial COI and COII genes revealed substantially higher haplotype diversity and finer-scale population differentiation. COI, in particular, displayed exceptional haplotype richness (Hd > 0.9) (S1 Table), consistent with its established sensitivity to recent demographic processes and maternal lineage tracking in mosquito vectors [2,34]. COII showed intermediate resolution, reinforcing its value as a complementary mitochondrial marker, as previously demonstrated in studies of *An. superpictus* and *An. culicifacies* complexes [16,17,35]. The combined use of nuclear and mitochondrial markers, therefore, provides a robust framework for resolving population structure and detecting cryptic genetic diversity in *An. tessellatus*.

### 4.3. Demographic history and haplotype structure

Across mitochondrial loci, Indonesian populations of *An. tessellatus* exhibit high haplotype diversity coupled with low nucleotide diversity, a genetic signature commonly associated with recent demographic expansion following historical bottlenecks [36]. Star-like haplotype networks observed in COI and COII analyses further support this interpretation, suggesting population growth from ancestral haplotypes with subsequent accumulation of localized mutations. Such demographic patterns have been reported in multiple mosquito species and are often linked to Pleistocene climatic oscillations, habitat expansion, or increased dispersal opportunities facilitated by anthropogenic environmental change [13,37]. In Indonesia, repeated cycles of sea-level fluctuation during the Pleistocene likely created alternating phases of population isolation and connectivity, shaping the observed mitochondrial diversity. However, the star-like network topology, characterized by short branches among multiple haplotypes, may also reflect recent divergence, ongoing gene flow, or demographic expansion; therefore, these interpretations should be considered with caution until formal demographic tests are conducted.

### 4.4. Biogeographic influences on lineage divergence

The geographic distribution of genetic lineages strongly reflects the complex biogeographic history of the Indonesian archipelago. The distinct clustering of Sulawesi populations across all markers is particularly notable and consistent with Sulawesi’s long-term tectonic isolation and composite geological origin [31,38]. Wallacean barriers have been shown to restrict gene flow in numerous taxa, and similar patterns of lineage divergence have been documented in insects, molluscs, and vertebrates [39,40]. Lineages associated with Sumatra and Java–Nusa Tenggara likely reflect historical fragmentation within Sundaland, combined with ecological heterogeneity and limited dispersal across marine barriers. Despite these isolating forces, genetic distances among Indonesian lineages remain below thresholds typically associated with fully reproductively isolated species, suggesting early-stage divergence rather than complete speciation. Although classical population-genetic statistics such as pairwise FSTF_{ST}FST, AMOVA, Mantel tests, and neutrality tests are commonly used in population-structure studies, we did not include them here because of uneven locality-level sampling, small sample sizes in several populations, and the combined use of nuclear and mitochondrial markers, which makes such analyses less robust and harder to interpret. Instead, we emphasize phylogenetic clustering, haplotype diversity, and sequence divergence as the most reliable indicators of structure in this dataset.

### 4.5. Implications for malaria and lymphatic filariasis control in Indonesia

From a public health perspective, the finding that *An. tessellatus* across Indonesia forms a genetically connected but regionally structured population has several implications. First, the presence of multiple nuclear and mitochondrial lineages suggests that local populations may differ in ecological preferences (e.g., larval habitats, attitude, microclimate), host choice, and behavior (indoor vs. outdoor biting, peak biting times), which can influence their capacity to sustain residual malaria and lymphatic filariasis transmission under current control measures [2,41]. Second, high haplotype diversity raises the possibility that traits such as insecticide resistance, if they emerge, could spread regionally through movement of adults or passive transport, as observed in other *Anopheles* vectors [42].

Indonesia has committed to malaria elimination by 2030, and understanding the full spectrum of vector species and lineages, including secondary and potential vectors like *An. tessellatus,* is critical to achieving this goal [43,44]. In several parts of South and Southeast Asia, *An. tessellatus* has been documented as a principal or secondary vector of malaria and lymphatic filariasis, suggesting that misidentification or underestimation of its role could compromise surveillance and intervention strategies [2,41]. The genetic structure documented here provides a framework for prioritizing further entomological investigations, including blood-meal analysis, sporozoite detection, and insecticide susceptibility testing in key lineages (e.g., Sumatra and Sulawesi clusters) that exhibit elevated divergence and may represent functionally distinct vector populations.

### 4.6. Limitations and future directions

Several limitations should be acknowledged. The sampling, while geographically extensive, remains uneven, with some islands and provinces represented by relatively few specimens, potentially underestimating local diversity and missing intermediate haplotypes. The study relies on three loci, one nuclear and two mitochondrial, which, although widely used and informative for species delimitation and population genetics in *Anopheles,* provide limited resolution compared to genome-wide single-nucleotide polymorphism or whole-genome approaches [19,21,34]. In addition, because sampling was uneven and many localities were represented by only one specimen, we did not infer fine-scale population parameters at the locality level. Therefore, results are interpreted as broad phylogeographic patterns rather than definitive estimates of within-location population structure. Furthermore, no ecological, behavioral, or vector competence data were integrated here, limiting the ability to link genetic lineages to epidemiological roles directly. Future research should therefore combine high-throughput genomic sequencing with targeted ecological and entomological investigations to test whether the genetic clusters identified in this study correspond to distinct eco-ethological units. Including additional nuclear markers or reduced-representation genomic data, such as RADseq or UCEs, would improve power to detect fine-scale structure and gene flow patterns, as demonstrated in other Southeast Asian taxa, such as squirrels and ricefish [40,45]. Longitudinal sampling and integration of landscape and seascape features would further clarify how historical and contemporary barriers, such as deep ocean trenches and mountain ranges, shape the genetic architecture of *An. tessellatus* across Indonesia [46,47].

## 5. Conclusions

This study demonstrates that *Anopheles tessellatus* populations in Indonesia are genetically structured into multiple geographically associated lineages, despite forming a single monophyletic group. Analyses of nuclear ITS2 and mitochondrial COI and COII markers consistently reveal high haplotype diversity, low nucleotide divergence, and limited gene flow among major island groups due to isolation by distance. These genetic patterns reflect the complex biogeographic history of the Indonesian archipelago and suggest ongoing intraspecific diversification within *An. tessellatus*. These findings highlight the presence of cryptic genetic structure in a medically important mosquito species and underscore the value of multilocus molecular approaches for elucidating evolutionary processes in vector populations.

Populations from West Sumatra, Central, and North Sulawesi, and Central Kalimantan exhibited genetic distances and unique haplotypes that may represent incipiently divergent lineages within the *An. tessellatus* complex and warrant an integrative taxonomic and ecological investigation. These aspects are particularly important to study considering the recognized and potential role of *An. tessellatus* as a malaria and lymphatic filariasis vector in Indonesia and neighbouring countries. These findings reinforce the need to incorporate this species complex into national vector surveillance programs, insecticide-resistance monitoring, and elimination planning efforts.

## Supporting information

S1 Fig Phylogenetic analysis of the ITS2 sequences of the *Anopheles tessellatus* complex using the Bayesian analysis. (PDF)

S2 Fig Phylogenetic analysis of the COI sequences of the *Anopheles tessellatus* complex using the Bayesian analysis. (PDF)

S3 Fig Phylogenetic analysis of the COII sequences of the *Anopheles tessellatus* complex using the Bayesian analysis. (PDF)

S1 Table The differences in haplotype diversity, nucleotide diversity, and clustering patterns observed among the three genetic markers (ITS2, COI, and COII) highlight their varying levels of genetic resolution. (PDF)
