# Supplementary material for: Genetic variability and population structure within the Anopheles tessellatus complex (Theobald, 1901) in Indonesia using ITS2 nuclear and COI, COII mitochondrial sequences
Source: PLoS One. 2026 Aug 14;21(8):e0347730. doi: 10.1371/journal.pone.0347730 (PMC13475890; doi:10.1371/journal.pone.0347730)

Bayesian analysis -Phylogenetic tree *Anopheles tessellatus ITS2*

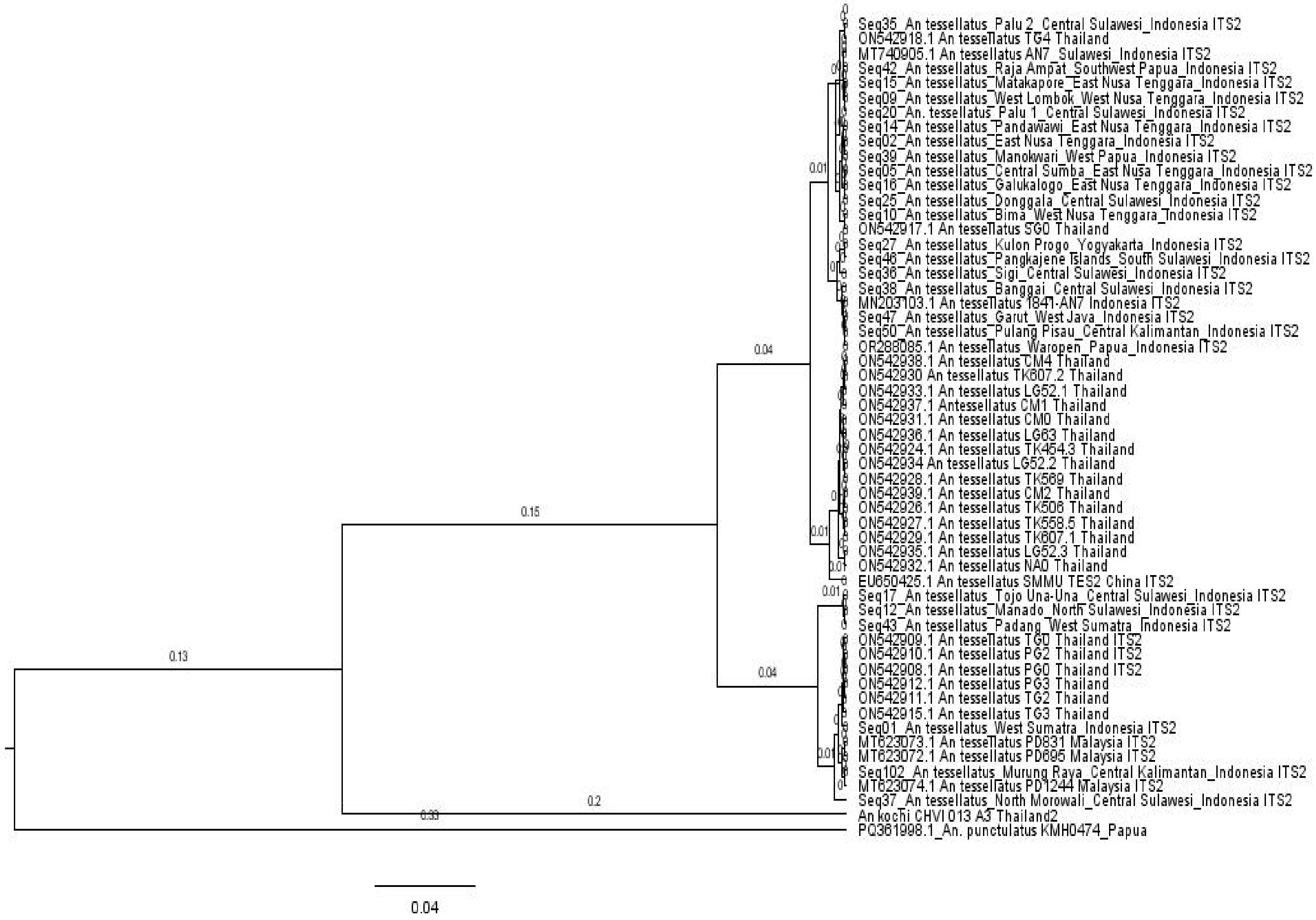

Supplement: Supplementary file 1 [file pone.0347730.s001.pdf]
