# Supplementary material for: Genetic variability and population structure within the Anopheles tessellatus complex (Theobald, 1901) in Indonesia using ITS2 nuclear and COI, COII mitochondrial sequences
Source: PLoS One. 2026 Aug 14;21(8):e0347730. doi: 10.1371/journal.pone.0347730 (PMC13475890; doi:10.1371/journal.pone.0347730)
Supplement: Supplementary file 4 [file pone.0347730.s004.pdf]

Marker	No. of haplotypes	Haplotype diversity (Hd)	Nucleotide diversity (π)	Main interpretation
ITS2	5	0.716	0.030	Lowest diversity; suggests more limited intraspecific variation and stronger conservation than the mitochondrial markers.
COI	35	0.914	0,039	Highest diversity indicates stronger phylogeographic resolution and more extensive population structure.
COII	17	0.931	0.034	High diversity; supports clear geographic structuring with substantial maternal-lineage variation.

Across the three markers, ITS2 showed the lowest genetic diversity, whereas COI and COII revealed much higher haplotype diversity and clearer phylogeographic structure. This pattern indicates that the nuclear ITS2 marker is more conserved and therefore less sensitive to recent population differentiation, while the mitochondrial markers better capture maternal lineage divergence and regional connectivity across Indonesian and broader Asian populations. In particular, COI displayed the broadest haplotype range, and COII showed similarly high diversity, suggesting that both mitochondrial loci are more informative for resolving fine-scale population structure and dispersal history in *Anopheles tessellatus*
